# Somatosensory input drives membrane potential dynamics in motor cortex during voluntary limb movement

**DOI:** 10.1371/journal.pbio.3003749

**Published:** 2026-04-17

**Authors:** Birgit C. Voigt, Florian Rau, Luc Estebanez, James F. A. Poulet

**Affiliations:** 1 Max Delbrück Center for Molecular Medicine in the Helmholtz Association (MDC), Berlin, Germany; 2 Neuroscience Research Center, Charité-Universitätsmedizin Berlin, Berlin, Germany; 3 Université Paris-Saclay, CNRS, Institut des Neurosciences Paris-Saclay, Saclay, France; The University of Edinburgh, UNITED KINGDOM OF GREAT BRITAIN AND NORTHERN IRELAND

## Abstract

How the motor cortex controls movement remains a fundamental question in neuroscience. Although somatosensory input is thought to influence motor cortex activity and the execution of voluntary movements, its role in driving motor cortex activity during voluntary behavior remains unclear. To address this, we performed whole-cell recordings from motor cortex neurons in mice during self-initiated, voluntary forelimb movements, either with intact somatosensory input or transection of the sensory nerves innervating the forelimb. In the absence of somatosensation, mice were still able to perform forelimb movements, including reaches, but these movements were significantly slower and more prolonged. Membrane potential recordings showed that cortical state changes were centrally generated, whereas external somatosensory input drives motor cortical activity before movement onset, curtails synaptic input during reaching to a hyperpolarized reversal potential value, and shapes membrane potential dynamics correlated with limb kinematics. Together, these findings demonstrate that somatosensory inputs play a central role in shaping motor cortex activity and its control of limb movement.

## Abstract 2

How does somatosensory input to the brain’s motor cortex contribute to limb movement? This study in mice shows that loss of somatosensory inputs disrupts voluntary forelimb movements and motor cortex neuronal dynamics, highlighting the role of somatosensory input in motor control.

## Introduction

Internal motor signals drive limb movements that enable interactions with the environment through actions such as reaching and touching objects. These movements trigger externally generated somatosensory inputs, conveyed by primary sensory afferent neurons innervating muscles, skin, and bones, which are relayed to motor centers and contribute to motor control by updating and fine-tuning ongoing movements. In support of this model, removal of somatosensory input is known to impair motor actions [1–8]. However, beyond its established role in corrective feedback, how somatosensory input drives neuronal activity in higher motor centers during voluntary movement remains unclear.

Motor cortex (M1) is an ideal region to address this question, as it plays a central role in voluntary motor control and is considered to be a key hub for integrating sensory and motor signals. In support of a role for somatosensory input to M1, anatomically well-defined somatosensory regions, both in the thalamus [9–14] and cortex [15–19], directly innervate M1. Functionally, M1 neurons show time locked responses to tactile stimulation of the forelimb [20–25] and proprioceptive-like responses to passive movement of the forelimb [23,26,27]. Theoretical models further emphasize diverse roles for somatosensory input in shaping M1 activity [28,29]. For example, predictive coding frameworks propose that M1 encodes predictions of expected proprioceptive sensory input [30], while optimal feedback control proposes that motor output is continuously refined by rapidly changing sensory inputs to achieve precise movement execution [31–33]. Together, these findings suggest that somatosensory inputs may drive synaptic and spiking activity in M1 neurons during limb movement, however, this hypothesis has not been directly tested experimentally.

M1 neurons show activity correlated with distinct phases of limb movement. Notably, M1 activity can precede movement onset [34–43] during both spontaneous and cued movements [44]. Moreover, during movement execution, the spiking activity of M1 neurons correlates with various aspects of limb kinematics [45–47], as well as kinetics and muscle-level features [48,49]. In addition, membrane potential and local field potential recordings from mouse M1 neurons during locomotion show prominent state changes, transitioning from slower, synchronized oscillatory activity during rest to faster, desynchronized fluctuations during movement [50,51]. The extent to which these distinct patterns of M1 activity are driven by somatosensory input is unknown.

In this study, we examine the contribution of somatosensory input to membrane potential dynamics in M1 during forelimb behavior. We compared M1 activity in control mice with that in mice in which somatosensory nerves innervating the forelimb were transected. Our data demonstrate that somatosensory input is essential both for the precise control of limb movement and for the regulation of M1 activity.

## Results

### Impact of forelimb somatosensory input on a self-paced, voluntary forelimb movement

**Fig 1:**
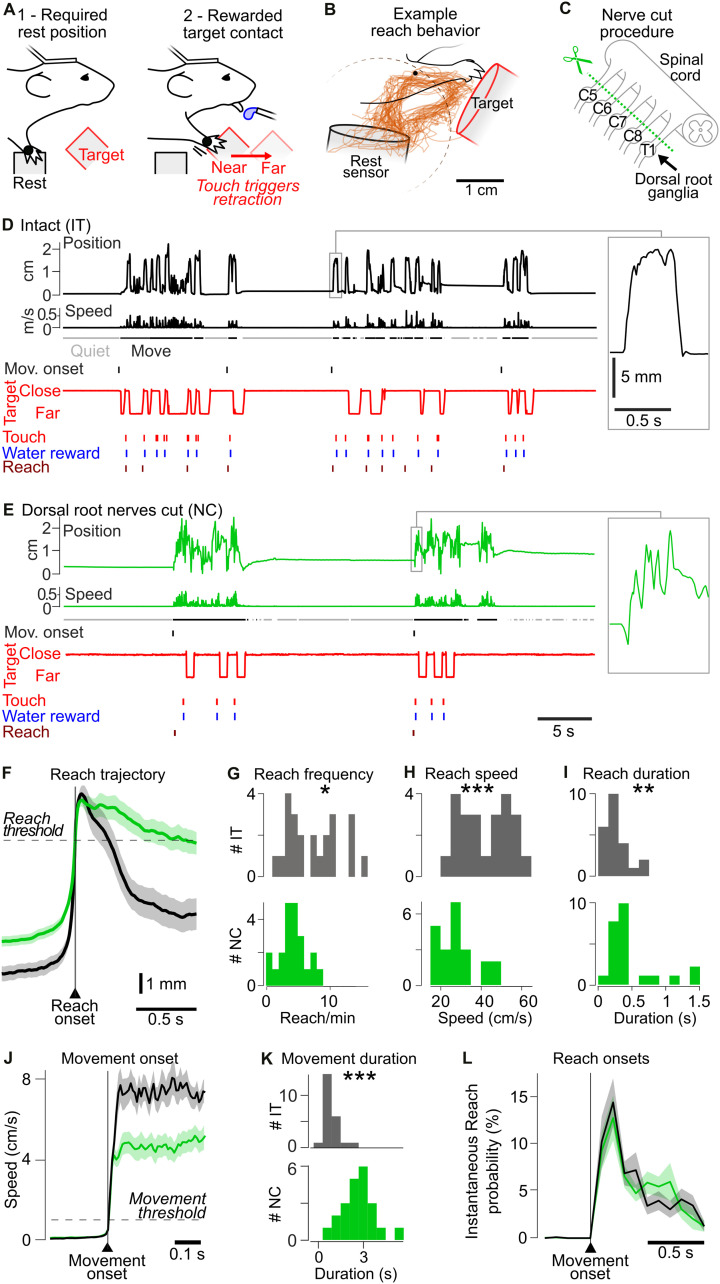
Impact of the removal of forelimb somatosensory feedback on forelimb reaching. **(A)** Cartoon schematic showing mouse forelimb reaching behavior. **(B)** Reach trajectories from an example mouse with forelimb somatosensory nerves intact (IT) (*n* = 36). Dashed circle: 1 cm threshold used to detect a reach (see panel F). **(C)** Schematic of dorsal root nerves transected in nerve cut (NC) mice. **(D)** Example behavior of an ITmouse. Left: From top to bottom: distance of the forelimb to the rest sensor; speed of forelimb movement; Quiet (gray) and Move (black) periods; time of the onset of Move periods; time of reach onset; and time of touch of the forelimb on the target sensor. Right: Zoom of an example reach. **(E)** Same as (D) but for a NC mouse. **(F)** Average distance of the forelimb from the rest sensor during a reach. Black: IT (*n* = 23 recordings from 22 mice). Green: NC (*n* = 22 recordings from 14 mice). Light background: SEM. Horizontal line shows the 1 cm distance threshold used to detect reaches. **(G)** Histogram of frequency of reach events in IT (gray) and NC (green) mice. *: Mann–Whitney *p* = 0.0129. **(H)** Histogram of average peak forelimb movement speed of individual mice during a 200 ms window centered on the Reach onset in IT and NC mice. ***: Mann–Whitney *p* = 0.00032. **(I)** Histogram showing difference in the distribution of the mean duration of reaches (time spent 1 cm from the rest sensor), in IT and NC mice. ** Mann–Whitney *p* = 0.0050. **(J)** Average time course of the speed of forelimb movement around the start of Move period. Light background: SEM across mice. **(K)** Histogram showing the longer duration of movement periods in NC than IT mice. ***: Mann–Whitney *p* = 4.4 × 10^−7^. **(L)** Instantaneous reach probability, showing a similar timing of the distribution of reaches following the onset of a movement sequence in IT and NC mice. Light background: SEM. The data underlying this Figure panels GHIK can be found in S1 Data.

To assess the role of somatosensory inputs on M1 activity during limb movement, we developed a forelimb reaching task for head-fixed mice. Water-restricted mice were trained to place their right forelimb on a rest platform that contained a capacitive contact sensor and to extend the forelimb towards a target sensor (Fig 1A, see methods). Touching the target sensor caused it to retract and trigger the release of a drop of water from a lick spout. Following training, mice performed reaches (7.46 ± 4.02 reach/min) as well as other self-paced forelimb movements, organized into bouts of movement periods interspaced by quiet, resting periods (Fig 1B). Reaches were defined as movements in which the forepaw remained within 1 cm of the rest sensor for at least 1 s, followed by a movement that crossed the 1 cm distance threshold (Fig 1B, Fig 1D and 1E).

Throughout the study, we compared M1 activity in mice with intact forelimb sensory nerves (intact, IT mice, *n* = 23) to mice subjected to a dorsal rhizotomy, in which the C5-T1 somatosensory dorsal roots innervating the right forelimb were severed (nerve-cut, NC mice, *n* = 14, Fig 1C). This manipulation is expected to remove the vast majority of somatosensory afferent input from the right forelimb [52], while preserving the motor efferent output via the ventral roots. In a subset of mice, we verified the effectiveness of the nerve transection by assessing cortical responses to forepaw vibrotactile stimulation using intrinsic optical imaging. In IT mice, forelimb stimulation evoked a robust activation of S1, whereas this response was absent following nerve transection (S1 Fig).

We next examined the impact of cutting the sensory nerves on forelimb behavior. Forelimb kinematics were quantified by tracking the absolute distance of a reflective marker on the forelimb relative to the rest position. Despite the loss of somatosensory input, NC mice were still able to perform forelimb movements, including reaches (Fig 1E and 1F), but there were significant differences compared to IT mice. For example, reaches were less frequent in NC mice (NC, 4.3 ± 0.44) compared to IT mice (7.2 ± 0.83 reach/min, Fig 1G). In addition, the forelimb rested in a more protracted position (S2A and S2B Fig). Moreover, peak forelimb speed during reaching was reduced in NC mice (Fig 1H, 28.5 ± 2.00 cm/s) compared with IT mice (42.4 ± 2.64 cm/s), and reaches had a longer duration (Fig 1I, NC, 0.48 ± 0.081 s, versus IT, 0.26 ± 0.038 s). In contrast, other parameters, including reach distance and amplitude as well as the positive correlation between speed and amplitude, remained similar between groups (S2F–S2H Fig).

Both sets of mice alternated between periods of active forelimb movement (Move), which included reaches as well as smaller forelimb movements, and resting (Quiet) periods with minimal movement (Fig 1D). To detect transitions between these behavioral states, we used a threshold of the forelimb movement speed (methods, Fig 1J). Quiet periods were shorter in NC mice (S2C Fig), whereas Move periods were significantly longer (Fig 1K). Although slow, subthreshold drift movements during Quiet periods were more frequently observed in NC than IT mice (S2A and S2D Fig), forelimb movement speeds were similar during movement (S2E Fig). NC and IT mice showed similar timing of reaches relative to the onset of movement bouts, with the majority of reaches occurring within the first second after movement onset (Fig 1L).

Thus, despite the loss of somatosensory input, NC mice were able to generate voluntary forelimb movements, including reaches, but these were slower and longer in duration.

### Forelimb motor cortex is involved in voluntary forelimb behavior

Next, we tested the causal role of forelimb M1 in our behavior. To locate forelimb M1, we performed electrical microstimulation in anesthetized mice (S3 Fig). Consistent with previous studies [53,54], microstimulation evoked movements in two regions: a caudal forelimb area (CFA), and a larger rostral forelimb area (RFA) of M1 (S3A Fig). Intrinsic optical imaging of forelimb somatosensory responses in primary forelimb somatosensory cortex (fS1) revealed that CFA is located in a more medial and anterior position relative to fS1, as previously observed [21,54] (S3A Fig).

To test whether the CFA was required for forelimb behavior during our task, we trained five mice to perform the reaching task and then pharmacologically inhibited CFA by local injection of the GABAergic agonist muscimol. In agreement with previous work [21,34,55], inhibition of CFA led to a significant reduction in the amount of reaches and general forelimb movements (S3B–S3D Fig). These effects were not observed following muscimol injection in auditory cortex or after Ringer’s injection in M1 (S3B–S3D Fig). Notably, we observed head-fixed face grooming following M1 muscimol injection in 2 of the 5 mice (S3E Fig). Post hoc histological analysis confirmed that injections were accurately targeted to CFA (S4 Fig). Thus, these data suggest that the CFA is involved in the control of reaching.

### Somatosensory input drives pre-movement activity in motor cortex

Prior work has shown distinctive pre-movement activity in M1, including membrane potential (Vm) modulations that might indicate preparatory synaptic input from upstream motor structures [34,36], as well as firing rate modulations that are thought to be directly involved in upcoming movement preparation [36–38,42,43,56]. One dominant hypothesis proposes that pre-movement activity corresponds to an internal representation of upcoming movement that evolves within an M1 encoding subspace and does not directly drive downstream motor commands (null space, [57]).

We suggest an alternative, non-exclusive, hypothesis that pre-movement M1 activity is at least partially driven by somatosensory input to M1, triggered by early muscle activation preceding overt forelimb movement. This is supported by previous work showing that both proprioceptive [23,26,27] and tactile inputs [21] can strongly drive M1 neurons and may serve as a timing signal for movement initiation [34]. Ongoing proprioceptive input during stable limb posture prior to movement may also contribute to pre-movement activity in M1 via tonically active M1 neurons sensitive to proprioceptive input [26].

**Fig 2:**
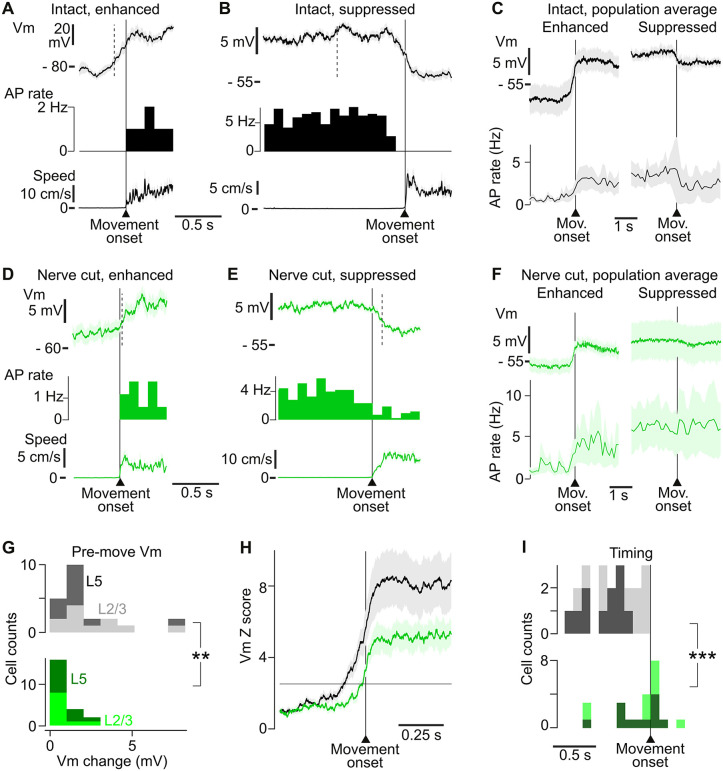
Somatosensory input drives pre-movement activity in M1 neurons. **(A)** Top: average Vm of an example IT-enhanced neuron around 10 movement onsets. Black shows mean, lighter background shows SEM. Middle: AP rate. Bottom: forelimb movement speed. Dashed vertical line represents the time of the first significant deviation of the Vm from the pre-movement baseline based on the Vm *Z* score (see panels H and I). **(B)** Same as (A) but for an IT-suppressed neuron (average around 34 movement onsets). **(C)** Average Vm (top) and AP rate (bottom) around movement onset, for all enhanced (*n* = 17) and suppressed (*n* = 5) neurons recorded in IT mice. See S7 Fig for further details about AP activity around movement. **(D)** Same as (A) but for a NC-enhanced neuron (17 movement onsets). **(E)** Same as (A) but for a NC-suppressed neuron (40 movement onsets). **(F)** Average Vm (top) and AP rate (bottom) around movement onset, for all enhanced (*n* = 17) and suppressed (*n* = 5) neurons in NC mice. **(G)** Distribution of the amplitude of Vm modulation during the pre-movement period, calculated as the absolute difference in the mean Vm in a 250 ms window immediately preceding movement onset (-0.25 to 0 s relative to movement onset) and a baseline period defined as a 500 ms window starting 1 s before movement onset (-1 to -0.5 s)L2/3 (light tone) and L5 (dark tone) neurons in IT mice (22 neurons) and NC mice (22 neurons). **: all IT vs. all NC, Mann–Whitney *p* = 0.0012. **(H)** Average *Z* score of the Vm (relative to a 1 s baseline window, 1 s before movement onset) in IT (black, *n* = 22) and NC (green, *n* = 22) neurons. Light background: SEM. Horizontal line: *Z* = 2.5 threshold. **(I)** Distribution of the onset times of movement-related Vm modulation, determined from panel (H) using the *Z* = 2.5 threshold. Light tone: layer 2/3. Dark tone: layer 5. ***: Mann–Whitney *p* = 0.00079 for all IT vs. NC neurons. The data underlying this Figure panels ABDEGI can be found in S1 Data.

To test this hypothesis, we performed whole-cell Vm recordings from M1 neurons in IT mice (*n* = 11 cells in Layer 2/3, 11 cells in Layer 5) and NC mice (*n* = 10 Layer 2/3 cells, 12 Layer 5 cells), with laminar identity confirmed by histology in a majority of cells (IT: *n* =16 cells, NC: *n* =19 cells, S5 Fig). We first analyzed the average Vm traces aligned to the start of Move periods, which included reaches as well as other voluntary forelimb movements. In IT mice, M1 neurons showed a rapid hyperpolarization or depolarization immediately prior to movement onset (Fig 2A–2C). In contrast, neurons recorded from NC mice showed little to no synaptic input prior to movement initiation (Fig 2D–2F). Quantification showed that the amplitude of pre-movement Vm modulation was significantly reduced in NC (0.63 ± 0.16 mV) compared to IT mice (2.03 ± 0.46 mV, Fig 2G).

To assess the timing of pre-movement input, we computed the *Z*-score of the Vm relative to baseline. In IT mice, pre-movement activity was different from baseline (*Z* score = 2.5 threshold, Fig 2H) significantly earlier than in NC mice (IT latency −0.45 ± 0.06 s, *n* = 22 cells, versus −0.15 ± 0.06 s *n* = 22 cells, Mann–Whitney *p* = 0.00079, Fig 2H and 2I). This difference remained even after excluding IT neurons with larger movement-related Vm modulations than those found in the NC population (S6F–S6H Fig), indicating that differences in pre-movement timing are not due to differences in the amplitude of Vm responses at or after movement onset. Moreover, we found no effect of the type of movement (reach versus non-reach, Mann–Whitney *p* = 0.14 in IT neurons, *p* = 0.69 in NC neurons) or of the speed of the movement (1–3 cm/s versus 4–6 cm/s movement speed groups, Mann–Whitney *p* = 0.50 for IT neurons, and *p* = 0.13 for NC neurons) on the timing of the pre-movement Vm. This indicates that the absence of pre-movement input in NC mice cannot be explained by differences in movement kinematics but instead is a result of the loss of somatosensory input to M1 prior to voluntary movement.

### M1 state changes are internally generated

Previous Vm recordings of cortical neurons have shown pronounced differences in the Vm dynamics during locomotion compared to stationary periods, defining transitions in cortical state [51,58–64]. To investigate whether somatosensory input contributes to state changes in M1, we compared Vm activity during Quiet and Move periods in IT and NC mice.

**Fig 3:**
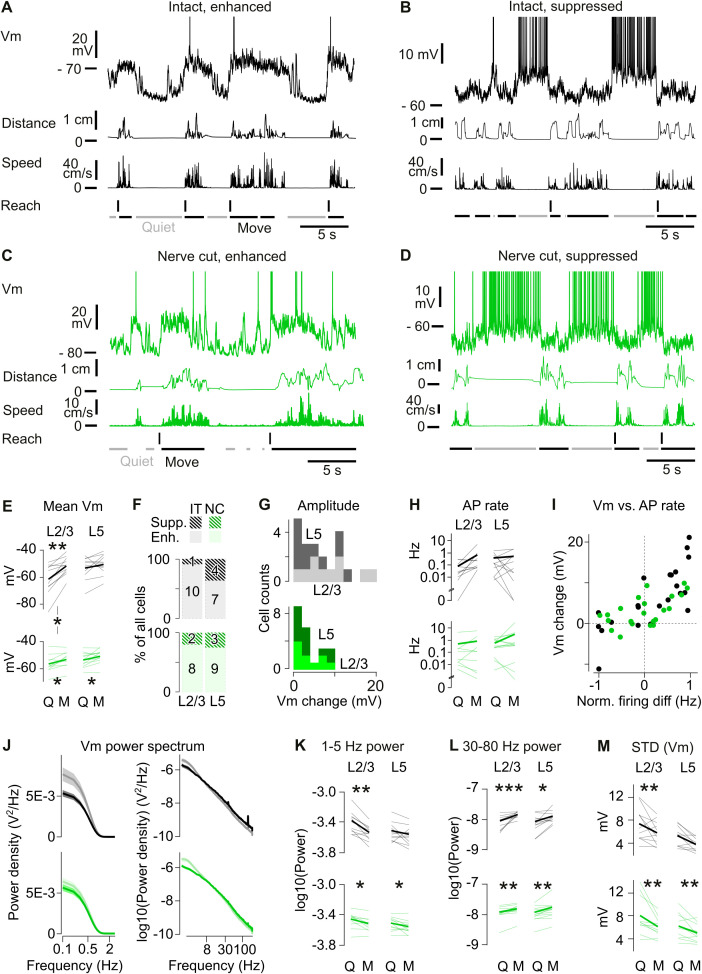
State-dependent changes in membrane potential activity of M1 neurons. **(A)** Top: Example Vm recording from an M1 neuron in an IT mouse showing depolarized, enhanced activity at movement onsets. Middle: right forelimb distance from rest position and instantaneous speed. Bottom: behavioral events based on forelimb movement. Vertical lines: reaches. Horizontal lines: Quiet and Move periods. **(B)** Same as (A), but for a suppressed neuron that hyperpolarizes during movement in an IT mouse. **(C)** Same as (A), but for an enhanced neuron in a NC mouse. **(D)** Same as (A), but for a suppressed neuron in a NC mouse. **(E)** Average Vm during Quiet vs. Move periods. Thin lines: single neuron average. Thick lines: population average. Top: IT mice. L2/3 **: Wilcoxon *p* = 0.0020. Bottom: NC mice. L2/3 *: *p* = 0.027. L5 *: *p* = 0.034. Comparison between NC and IT neurons in L2/3. Quiet: Mann–Whitney *p* = 0.022. Other NC vs. IT comparisons are not significant. **(F)** Proportion of enhanced neurons (filled color) and suppressed neurons (line-patterned) across layers and experimental conditions (IT vs. NC). Numbers on the bars: corresponding count of neurons. **(G)** Distribution of the absolute difference in Vm between Quiet and Move periods in L2/3 (light tone) and L5 neurons (dark tone). **(H)** Average firing rate during quiet and movement periods plotted on a log scale. Thin lines: single neuron average. Thick line: population average. No significant difference. **(I)** Change in Vm from Quiet to Move as a function of change in firing rate (normalized as the difference between the 2 conditions, divided by the sum). Pearson correlation coefficient *r* = 0.77 (IT), 0.61 (NC). **(J)** Power spectrum of the Vm of IT neurons during Quiet (gray) vs. Move periods (black), displayed as a function of frequency in logarithmic units. Top: IT neurons, Bottom: NC neurons. Quiet: light green. Movement: dark green. Right: same data in a log–log scale to highlight higher frequencies. **(K)** Power in the 1–5 Hz frequency band. Top: IT mice. L2/3 **: Wilcoxon *p* = 0.0020. Bottom: NC mice. L2/3 *: *p* = 0.027. L5 *: *p* = 0.027. **(L)** Power in the 30–80 Hz frequency band. Top: IT mice. L2/3 *** Wilcoxon *p* = 0.00099. Bottom: NC mice. L2/3 **: *p* = 0.0020. L5 **: *p* = 0.0068. **(M)** Standard deviation of the Vm during Quiet vs. Move periods. Top: IT mice. L2/3 **: Wilcoxon *p* = 0.0068. Bottom: NC mice. L2/3 **: *p* = 0.0059. L5 **: *p* = 0.0068. The data underlying this Figure panels EFGHKLM can be found in S1 Data.

In both groups, M1 neurons showed clear differences in synaptic activity between these two behavioral states (Fig 3A–3D). At the transition from Quiet to Move, M1 neurons either depolarized or hyperpolarized (Fig 3A–3E), with a greater proportion showing depolarization (enhanced neurons 77%) than hyperpolarization (suppressed neurons 23%). In both IT and NC mice, the majority of suppressed neurons were observed in L5 (Fig 3F). It was possible to histologically identify the cell type of a subset of neurons (11 WT and 15 NC recordings). All identified interneurons (3/3) were enhanced during Move periods, whereas of the identified pyramidal neurons most were enhanced (19/23) and few were suppressed (4/23) during movement.

The magnitude of the mean Vm difference between Quiet and Move periods varied across layers and groups. In IT mice, this difference was significantly larger in L2/3 (9.8 ± 2.1 mV) than in L5 neurons (2.7 ± 2.0 mV (Mann–Whitney *p* = 0.032, Fig 3G). In contrast, the difference in NC L2/3 neurons was similar to that observed in NC L5 neurons (average difference 3.3 ± 1.2 mV in L2/3, 2.7 ± 1.1 mV in L5). The Quiet-Move difference in IT L2/3 neurons was also significantly greater than that observed in NC L2/3 neurons (Mann–Whitney *p* = 0.022). The reduced Quiet-Move Vm modulation in NC L2/3 neurons might reflect a slightly more depolarized Vm during Quiet periods in NC (average −54.9 ± 1.3 mV) compared with IT mice (average −57.5 ± 2.2 mV), although this difference was not significant. However, changes in firing rate between Quiet and Move periods were not significantly different between IT and NC mice (Fig 3H), and showed a similar positive correlation with the underlying change in Vm (Fig 3I).

Similar differences were observed in the frequency content of ongoing Vm oscillatory activity between Quiet and Move periods in IT and NC mice (Fig 3J). Using FFT analysis, we found that M1 neurons showed a reduction in low-frequency 1–5 Hz modulations during movement both in IT and NC mice (Fig 3J and 3K). Consistent with this reduction in slow, large amplitude fluctuations, the standard deviation of Vm was significantly lower during movement in both groups (Fig 3M). In contrast, power in the 30–80 Hz band increased during Move periods in both IT and NC mice (Fig 3J and 3L).

The overall similarity of changes in state changes across IT and NC neurons suggests that the M1 state transitions are primarily driven by internally generated, non-sensory input. However, the slightly reduced amplitude of the Vm modulation in L2/3 neurons following removal of somatosensory inputs (Fig 3E) indicates that somatosensory input contributes a small, modulatory component to the state-dependent changes in M1 activity.

### Somatosensory input regulates the strength of reach responses in M1 neurons

Because somatosensory input plays a role in driving synaptic input preceding movement onset (Fig 2), we hypothesized that it also influences M1 neuronal modulations during voluntary reaching. To test this, we examined the synaptic and spiking activity of M1 neurons during trained reaches.

**Fig 4:**
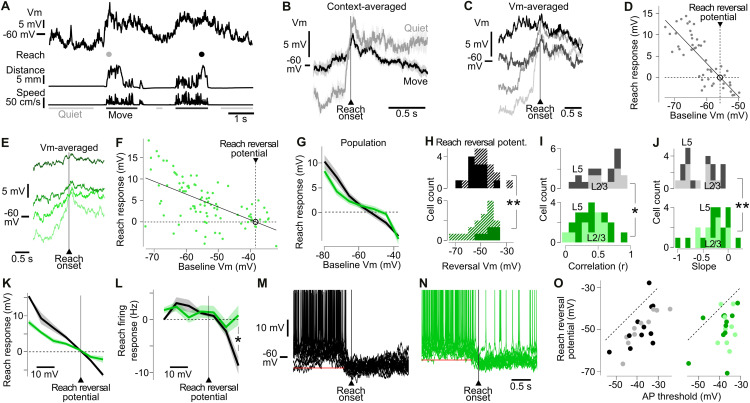
Somatosensory input drives hyperpolarized reversal potentials of reach-evoked postsynaptic potentials. **(A)** From top: Example Vm recording during reaching. Gray dot shows a reach following a quiet period at the onset of a movement period, black dot a reach during movement. **(B)** Averaged reach response for neuron in (B) following a Quiet (*n* = 8 trials, gray) or Move period (*n* = 33 trials, black). Light background: SEM. **(C)** Averaged reach response from the same neuron as in (B) at 4 equal categories of pre-reach baseline Vm, from the most depolarized baseline (black) to the most hyperpolarized (light gray). **(D)** Plot showing negative correlation between the reach response amplitude and the pre-reach baseline Vm, same neuron as in (B). Linear regression (*r* = −0.81). Black circle shows the reach reversal potential where there is no change in Vm during a reach. **(E, F)** Same as (D, E) but for a NC mouse (*r* = −0.60) showing a more depolarized reach reversal potential. **(G)** Population average Reach response plotted as a function of the baseline Vm in IT (black) and NC mice (green). **(H)** Distribution of the reach reversal potential in IT (top) and NC (bottom) neurons. Plain color-filled histograms show distribution for neurons where the linear regression between baseline Vm and reach response explained more than 30% of the variance (*r* > 0.55). Among these neurons, the reach reversal was significantly larger in NC neurons than in IT neurons. **: Mann–Whitney *p* = 0.0097. Diagonal line fill: neurons with *r* ≤ 0.55. **(I)** Population distribution of the correlation between baseline Vm and the reach response amplitude. At the population level, the correlation is significantly smaller in NC than in IT mice. *: all layers, Mann–Whitney *p* = 0.016. Light tones: L2/3 (Mann–Whitney *p* = 0.018). Dark: L5 (Mann–Whitney *p* = 0.41). **(J)** Population distribution of the slope of the linear correlation between baseline Vm and reach response amplitude (** all layers, Mann–Whitney *p* = 0.0072). Light tones: L2/3 (Mann–Whitney *p* = 0.018). Dark: L5 (Mann–Whitney *p* = 0.15). **(O)** Reach reversal potential plotted as a function of the AP threshold. Left: IT mice (all layers Pearson *r* = 0.73, *p* = 2 × 10^−4^, L2/3: *r* = 0.89, *p* = 5 × 10^−4^, L5: *r* = 0.59, *p* = 0.055). Right: NC mice (all layers Pearson *r* = 0.35, *p* = 0.12, L2/3: *r* = 0.02, *p* = 0.96, L5: *r* = 0.45, *p*= 0.14). **(K)** Average reach response amplitude as a function of the baseline Vm in IT (black) and NC mice (green). The reach reversal potential was subtracted from Vm in this graph. **(L)** Same as (K) but for firing rate. For baseline Vm values above the reach reversal potential, the firing rate change upon reach becomes significantly more negative in IT than in NC mice. *: Mann–Whitney *p* = 0.0152. **(M)** Example Vm traces during a reach for a suppressed neuron in an IT mouse (67 reaches). Red line: reach reversal potential for this neuron. **(N)** Same as (M) but for *n* = 43 reaches in a NC mouse. **(O)** Reach reversal potential plotted as a function of the AP threshold. Left: IT mice (all layers Pearson *r* = 0.73, *p* = 2 × 10^−4^, L2/3: *r* = 0.89, *p*= 5 × 10^−4^, L5: *r* = 0.59, *p*= 0.055). Right: NC mice (all layers Pearson *r* = 0.35, *p* = 0.12, L2/3: *r* = 0.02, *p* = 0.96, L5: *r* = 0.45, *p*= 0.14). Light tone: L2/3, dark: L5. The data underlying this Figure panels HIJ can be found in S1 Data.

The average change in action potential (AP) firing rate during reaches was low both in IT mice (+0.39 Hz) and NC mice (−0.13 Hz). To examine the synaptic mechanisms underlying this sparse firing, we averaged reach-triggered Vm responses during Quiet and Move periods (Fig 4A). In IT mice, reach-evoked responses were larger during hyperpolarized Quiet periods than during depolarized Move periods, despite similar reach kinematics (S2I–S2K Fig). This indicates that baseline Vm strongly modulates synaptic input during reaching (Fig 4B). In support of this hypothesis, grouping reach responses by pre-reach Vm showed a decrease in response amplitude with increasing Vm (Fig 4C). Plotting individual reach responses against pre-reach Vm showed a strong and significant negative correlation that crossed 0 mV amplitude near −55 mV (Fig 4D). We termed this value the “reach reversal potential.” Because the reversal potential for glutamate is around 0 mV, the more hyperpolarized reach reversal potential indicates a major contribution of GABA-ergic inhibition to reach-evoked synaptic responses, which may help explain the sparse firing rates observed during reaching.

The reach reversal potential differed significantly between groups. In IT mice it was −52.0 mV, whereas it was −43.9 mV in NC mice (Mann–Whitney *p* = 0.0097, recordings with *r*^2^ > 30%, Fig 4E–4H). Moreover, the correlation between reach response amplitude and pre-reach Vm was weaker in NC mice, and the slope of the correlation significantly shallower (Fig 4I, 4J, and 4K). Together, these finding suggest that somatosensory input shifts the reach reversal potential to more hyperpolarized values. One possibility is that somatosensory input recruits GABA-ergic inhibitory neurons which inhibit local excitatory neurons during reaching.

To relate these synaptic effects to spiking output, we plotted the reach-evoked firing response against the pre-reach baseline Vm (Fig 4L). This revealed a distinct relationship between firing rate and pre-reach baseline Vm in IT compared to NC neurons. Notably, the reach evoked spike rate of NC neurons was significantly higher at the most depolarized pre-reach values (Fig 4L), consistent with reduced synaptic inhibition following loss of somatosensory input. This effect was particularly evident in suppressed neurons, cells with a depolarized baseline Vm that stopped firing during movement in IT mice, but less so in NC mice (Fig 4M and 4N).

Finally, we examined how removal of somatosensory input affected the relationship between the reach reversal potential and the AP threshold. Although AP thresholds were varied across cells, in both NC and IT neurons, the reach reversal potentials were significantly more hyperpolarized than the AP threshold (reversal: −48.9 ± 1.9 mV in IT, −49.2 ± 1.9 mV in NC; AP threshold: −37.9 ± 1.4 mV in IT, −37.7 ± 1.1 mV in NC, Wilcoxon *p* = 9 × 10^−5^, Fig 4O). In IT, but not NC mice, the reach reversal potential was positively correlated with AP threshold, indicating a role for somatosensory input in regulating the intrinsic excitability of M1 neurons. These effects were layer-specific. The level of correlation and slope of the relationship between baseline Vm and reach response was significantly impacted by the nerve cut in L2/3 neurons but not in L5 (Fig 4I and 4J). Similarly, the relation between AP threshold and reach reversal potential was also significant in L2/3 IT neurons, and disrupted by the nerve cutting, while it was not significant in IT L5 neurons (Fig 4O). Given the known role of M1 L2/3 in processing somatosensory inputs [17,24], these observations further support the conclusion that somatosensory inputs play a key role in the regulation of reach-related Vm dynamics and cellular excitability in M1.

### Limb movement-correlated Vm modulations are driven by somatosensory input

Our previous analyses examined M1 activity at discrete movement onset-triggered events. However, during ongoing, sustained limb movements, M1 activity is known to be correlated with different kinematic parameters [45–47]. Whether somatosensory input contributes to the correlation between ongoing limb kinematics and the Vm dynamics of M1 neurons has not been examined.

**Fig 5:**
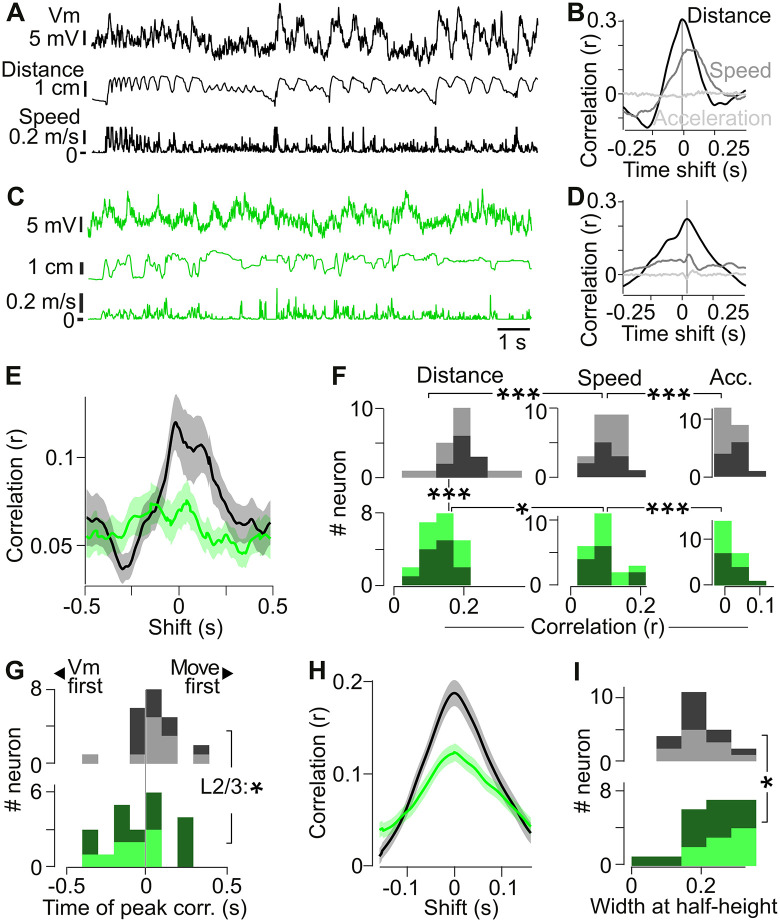
Somatosensory feedback shapes membrane potential correlations with limb movements. **(A)** Example Vm recording from an IT mouse during a movement period. Top: Vm high-pass filtered at 0.2 Hz. Middle: simultaneously recorded distance of the forelimb from the rest sensor. Bottom: forelimb movement speed. **(B)** Cross-correlations for the data in (A) between the Vm and kinematic parameters: the distance to the rest sensor (black), the speed (dark gray), and acceleration (light gray). Vertical line indicates the time of the peak Vm-distance cross-correlation. **(C, D)** Same as for (A, B), but for an example NC mouse. **(E)** Population average of the absolute cross-correlation between forelimb distance and Vm. **(F)** Distribution of the peak absolute correlation values between the Vm and forelimb distance, speed and acceleration for IT mice (top) and NC mice (bottom). Light tone: L2/3 neurons; dark tone: L5 neurons. Correlation of Vm with distance is significantly larger than Vm with speed in IT (***: Wilcoxon *p* = 0.00048) and NC mice (*: *p* = 0.046). Correlation of speed with acceleration is significantly different in IT (***: *p* = 0.000040) and NC mice (***: *p* = 0.000040). Although Vm-distance correlations were significantly larger in IT than in NC mice (***: Mann–Whitney *p* = 0.00069), Vm correlations with speed and acceleration did not differ between groups (*p* > 0.05). **(G)** Timing of the peak absolute value of the cross-correlation between Vm and distance. In IT mice, movement precedes Vm (Wilcoxon *p* = 0.024). The difference in timing between IT and NC mice is significant in L2/3 neurons (*: Mann–Whitney *p* = 0.0226) but not in L5 neurons and not at population level. **(H)** Average absolute cross-correlation profile between Vm and distance in IT (black) and NC (green) mice, realigned to the peak to highlight differences in half-width. **(I)** Half-width of the Vm-distance cross-correlations in (H) is significantly larger in NC than in IT mice.*: Mann–Whitney *p* = 0.030. Light tone: L2/3. Dark tone: L5. The data underlying this Figure panels FGI can be found in S1 Data.

To address this, we calculated cross-correlations between the Vm and the distance moved, speed of movement, and acceleration during movement periods (Fig 5A and 5B). To minimize the contribution of state-dependent fluctuations in Vm, we high-pass filtered the Vm (<0.2 Hz), isolating faster Vm modulations during sustained bouts of movement.

In IT mice, cross-correlation analysis revealed that 95% of M1 neurons showed a correlation of Vm modulation with forelimb distance moved (peak *r* > 0.1, Fig 5E and 5F). In contrast, fewer neurons were correlated with forelimb speed (55%), and none with forelimb acceleration (no neuron reached *r* = 0.1, Fig 5F). Across the population, peak correlation values with distance were larger than those with speed which in turn were significantly larger than those with acceleration (Fig 5B and 5F). These results indicate that forelimb distance moved is a prominent movement parameter encoded in M1.

Neurons in NC mice also showed correlations between Vm and movement parameters (Fig 5C and 5D). Similar to IT neurons, peak correlations were largest with distance, followed by speed and then acceleration (Fig 5F). However, the proportion (*r* > 0.1 68% NC versus 95% in IT, proportion *Z* test *p* = 0.019) and strength of correlation of forelimb distance with Vm (Fig 5F) were significantly lower in NC than in IT mice. These differences were not due to differences in the power spectrum of forelimb distance between NC and IT mice (S6A–S6E Fig). Together, these data indicate a role for somatosensory input in driving the correlation between Vm and forelimb movement.

We next examined the temporal structure of the correlations. In some cells, the peak Vm-forelimb distance correlation occurred at time lags different from zero, indicating that Vm either followed movement (positive peak time) or preceded it (negative peak time). Across neurons with correlation values *r* > 0.05, Vm modulations in IT mice preceded forelimb movement by 56 ms, whereas in NC mice, Vm lagged behind movement by 43 ms (Fig 5G). This difference in timing was more pronounced in L2/3 neurons, where the average timing shift between IT and NC mice was 160 ms (Fig 5G), again suggesting a role for somatosensory input to L2/3.

The width of the cross correlation gives an indication of the temporal characteristics of the interaction between Vm and movement. We found that the Vm-distance correlation was significantly broader in NC than in IT mice (Fig 5H and 5I), even after equalizing the forelimb movement power spectrum between groups (Figs 5E and S6D). This broadening indicates that in the absence of somatosensory input the ability of M1 neurons to track fast, higher frequency forelimb movement is diminished.

Together these results suggest that externally generated somatosensory input is integrated with internal motor signals on a fast time scale to drive fast, precisely timed changes in Vm activity during limb movement. In the absence of this input, M1 neuronal dynamics become slower and less tightly coupled to forelimb movement kinematics.

### Somatosensory input drives fast reach and touch input to M1

**Fig 6:**
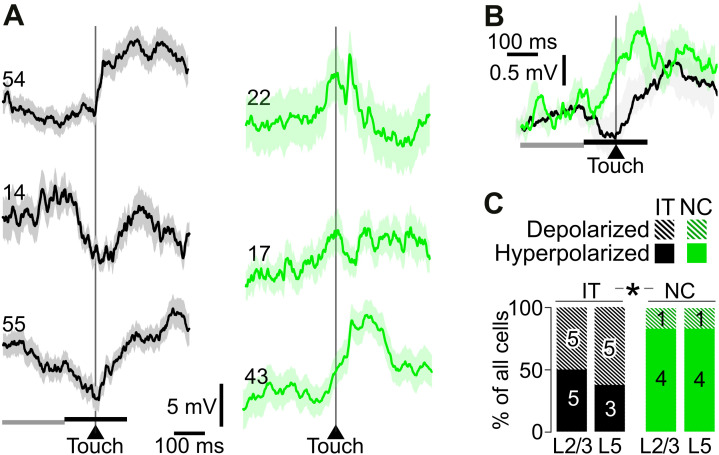
Somatosensory input drives a hyperpolarization during a touch. **(A)** Examples of 3 averaged Vm dynamics around touch time from IT (black) and NC mice (green). Light background: SEM. Examples show a significant modulation (Wilcoxon *p* < 0.05) of the average Vm between baseline (−300 to −100 ms prior to touch, gray horizontal line) and touch time (−100 to +100 ms around touch, black horizontal line). Numbers on traces show the  numbers of reach and touches per cell average. **(B)** Population average Vm of all IT (black, *n* = 18) and NC (green, *n* = 10) neurons around touch time. Light background: SEM. Horizontal black lines: baseline and touch-aligned measurement used for the population analysis in C. **(C)** Proportion of cells that show touch-related Vm depolarization from baseline vs. a hyperpolarization in IT compared to NC mice. Numbers on bars show the corresponding neuron counts. *: significant difference in proportion of depolarizing vs. hyperpolarizing neurons in the WT vs. NC categories (layers merged). *Z* test *p* = 0.034. The data underlying this Figure panel C can be found in S1 Data.

One potential source of the fast correlations between Vm and limb movements could be proprioceptive or tactile input generated during reaching and contact with the target sensor. To test this, we averaged the Vm of M1 neurons in IT mice at the time of touch with the target sensor. This revealed significant Vm modulations that varied in timing around the touch time (Fig 6A, left).

Across the population, touch was associated with an overall hyperpolarizing response around the touch time in IT mice (Fig 6B, black trace). This hyperpolarization occurred in the context of the broader Vm depolarization around movement and reach onset. Although these modulations could result from somatosensory input, they might also be evoked by internally generated motor signals or predictive signals relating to the expectation of a touch or water reward. To isolate the role of somatosensory feedback, we compared these responses in IT with those in NC mice.

In NC mice, M1 neurons also showed Vm modulations around touch time. However, in contrast to the overall hyperpolarizing response in IT mice, the population average response was a depolarization (Fig 6B, green trace). To quantify this difference, we classified neurons based on the sign of the Vm change in a 200 ms window centered around touch compared to a 200 ms window −300 to −100 ms prior to touch. This analysis revealed that the proportion of neurons showing a hyperpolarization around touch was significantly smaller in NC compared to IT mice, with no significant difference between L2/3 and L5 (Fig 6C).

These data indicate that the fast correlations between forelimb movement and Vm (Fig 5) are in part driven by somatosensory input. Moreover, in agreement with inhibitory somatosensory input shaping M1 activity during reaching (Fig 4), reach and touch evokes fast, hyperpolarizing inputs to M1 neurons. These findings suggest that somatosensory feedback provides fast inhibitory input to M1 during reaching that shapes Vm dynamics.

## Discussion

**Fig 7:**
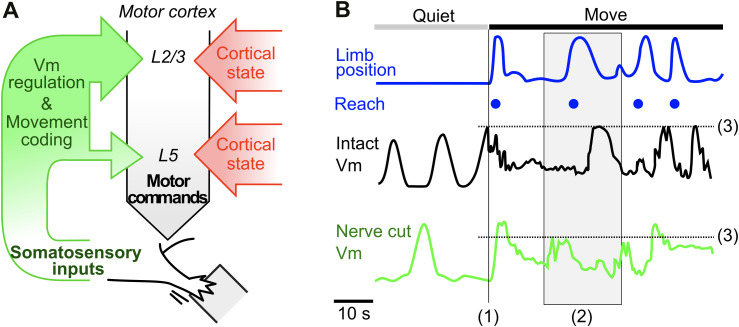
Summary of main findings. **(A)** Main contributions of the somatosensory inputs to the generation of motor activity in M1. Cortical state generation is independent from somatosensory inputs. **(B)** Schematic Vm modulations of an M1 neuron in an intact mouse (IT Vm) and in a nerve cut mouse (NC Vm) during a Quiet to Movement transition. The removal of forelimb inputs did not impact the occurrence of quiet/active periods in the membrane potential, but (1, vertical line) reduced pre-movement input; (2, gray box) reduced Vm correlations with ongoing forelimb movement; and (3, horizontal line) disrupted the reach reversal potential.

In this study, we investigated how somatosensory input shapes M1 activity during voluntary forelimb movements. Whole-cell recordings in behaving mice revealed two major interacting components, a centrally generated M1 state change and a faster somatosensory-driven input that act together to regulate M1 Vm dynamics before and during forelimb movements. Together these inputs participate in movement encoding and regulate synaptic integration and spiking in M1 neurons (Fig 7).

### Forelimb movement without somatosensory feedback

A direct approach to examine the impact of sensory information on motor encoding is the reduction or removal of the sensory input. Because somatosensory input is transmitted via mechanical deformation of the skin or muscle movement, experimentally reducing feedback in the somatosensory system is technically challenging. We addressed this by performing a dorsal rhizotomy in trained mice, which deafferented the central nervous system from right forelimb somatosensory input.

Despite the established role of somatosensory feedback in mammalian forelimb motor control, deafferented mice retained the ability to perform voluntary forelimb reaches and non-reach movements. However, their motor behavior was altered significantly. Compared to control mice, general forelimb movements occurred more frequently and lasted longer, while reaches became less frequent, slower and of longer duration. Our findings resemble observations in other species. Humans with a somatosensory neuropathy show impairments during planar reaching task including errors in movement direction, velocity, and acceleration and movements tend to be of longer duration and lack precise endpoints [65,66]. Likewise, monkeys with an acute dorsal rhizotomy exhibit ataxia and an inability to perform grasping movements with the fingers and hand while capable of performing rudimentary, inaccurate, forelimb movements [5,67,68]. Together with these classic findings, our results support the conclusion that somatosensory feedback is not absolutely required for motor output generation, but is critical for shaping the timing and structure of forelimb movements. Whether these changes are due to a lack of perception of the forelimb causing voluntary changes in movement strategy, or the result of disruption of subcortical motor neuronal dynamics remains to be determined.

The reduction in somatosensory input to M1 most likely reflects a reduction in sensory driven input via the somatosensory thalamus and/or primary somatosensory cortex. However, lesion-based manipulations may have induced compensatory plasticity within different sensory pathways or disrupted the balance of neuronal dynamics in higher-order or motor circuits that provide input to M1. For example, motor circuits in the basal ganglia and the cerebellum integrate somatosensory inputs and may then operate outside of their physiological range following deafferentation [69,70]. Another concern is that the surgery may have altered forelimb biomechanics, thereby changing movement kinematics and resulting in mice adopting alternate forelimb control strategies. In addition, the loss of forelimb somatosensory feedback and the invasive nature of the surgery may have altered internal states, including general arousal, possibly affecting our recordings in a non-specific manner. Future studies incorporating sham surgeries, reversible and temporally precise manipulations of somatosensory feedback during movement, and direct measurements of muscle tension and force, will be important to address these limitations. They may also help further disentangle the loss of external somatosensory input from centrally generated input to M1, and understand the sensorimotor circuitry underlying M1 function.

### Somatosensory input contributes to sub-threshold pre-movement M1 activity

In agreement with prior studies [34–37,40,42,43,71], we observed M1 activity preceding forelimb movements (Figs 2 and 6). Intriguingly, whole-cell recordings showed that pre-movement synaptic input was reduced in amplitude and occurred closer to movement onset following deafferentation. We therefore hypothesize that pre-movement activity in M1 is likely driven by somatosensory inputs, for example, those triggered by tonic somatosensory inputs arising from the static posture of the limb before movement onset [26,27], preparatory muscle activity, and/or by small postural changes immediately prior to movement onset (S1 Fig, [72]). Fast synaptic input to M1 has also been observed during a cue-triggered lever pressing, where cerebellar output reaches M1 via the motor thalamus [34], raising the possibility that somatosensory signals might be relayed to M1 via this pathway and contribute to movement initiation.

Previous work in monkey M1 reported that the ablation of the forelimb sensory branch did not impact the timing of the preparatory spiking activity prior to forelimb reaching [73,74]. In contrast, our whole-cell recordings showed that pre-movement synaptic input was reduced in amplitude and closer to movement onset following deafferentation. Differences in task structure, sensory cues, recording method and the duration of recovery periods following deafferentation make it hard to identify the source of these differences. However, in some example neurons, we observed pre-movement spiking following nerve cutting (S7 Fig), suggesting there may be a subset of cells with non-sensory pre-movement input.

Because deafferented monkeys show movement errors at reach onset, it has been suggested that somatosensory input is not only involved in the online correction of ongoing movements, but also at early planning stages of upcoming movements [65,66]. Whether pre-movement synaptic somatosensory input encodes specific features of an upcoming limb movement remains an open question and could be tested in future studies where mice have been trained to perform directional reaching.

### Motor cortex state is centrally generated

We observed a prominent change in the state of M1 neuronal activity in behaving animals (Fig 3). Specifically, low-frequency, large-amplitude Vm fluctuations during rest were replaced by higher-frequency, smaller-amplitude inputs during movement. These changes in synaptic dynamics were accompanied by changes in the mean Vm value and the AP firing rate. Most neurons showed a depolarization and increased firing during periods of limb movements, but a subset of L5 neurons hyperpolarized and showed a suppression of firing.

This change in state was evident during both reaching and non-reaching movements and, together with similar changes in forelimb M1 during locomotion [34,51] and in forelimb primary somatosensory cortex during forelimb movement [64], suggests a general movement-related phenomenon rather than one specific to reaching. State dependent changes in synaptic input and AP firing persisted after cutting the forelimb somatosensory nerves suggesting a central origin. This mirrors similar findings in the primary somatosensory whisker cortex, where removal of sensory feedback from the whiskers does not abolish cortical state change during whisking [62].

### Somatosensory-evoked cortical inhibition determines sparse firing of M1 neurons during forelimb movement

Despite the large amplitude variability in Vm values prior to reach onset, the Vm consistently converged toward a specific absolute Vm value following each reach, which we termed the “reach reversal potential.” This value corresponds to the pre-reach Vm at which the reach evoked response produces no net change in voltage. When the pre-reach value is more depolarized than this value the reach induces a hyperpolarization and if more hyperpolarized would evoke a depolarization. This is a similar concept to the “sensory reversal potential” described in somatosensory cortex following a tactile stimulus [64,75].

In IT mice, the reach reversal potential was about −40 mV and closely correlated with the AP threshold, typically remaining about 12 mV below it. Given that the reversal potential for cortical GABA-ergic inhibition is near −80 mV and near −0 mV for glutamate, a hyperpolarized reach-evoked reversal potential indicates a substantial role for cortical inhibition in regulating reach responses in M1. In support of this interpretation, electrophysiological and optical recordings of M1 GABAergic interneurons activity have shown phasic activation of different subtypes of interneurons during sensory evoked and spontaneous forelimb movements [21,76,77]. In this way, the cortical network could regulate firing rates during sensorimotor integration.

The regulation of the movement-related input to M1 was dependent on somatosensory inputs. In deafferented mice, the reach reversal potential was more depolarized and less correlated to the AP threshold. These effects were most prominent in L2/3 neurons, where the majority of S1 somatosensory projections terminate. Moreover, during a reach and touch movement, deafferentation reduced the proportion of neurons that hyperpolarized. Together these findings support the view that somatosensory input from the limb engages inhibitory neurons in M1 [24,78], which in turn constrains neuronal spiking during limb movement. A reduced level or a mistiming of cortical inhibition may therefore contribute to the dysregulation of limb movement observed following deafferentation. The specific cortical interneuron types responsible for generating the hyperpolarized reach reversal potential remain to be identified.

The presence of a hyperpolarized reach reversal potential mirrors the state dependent modulation of fast sensory responses in primary somatosensory cortices, where sensory response reversal potentials are also hyperpolarized and linearly correlated with baseline Vm [58,64,75,79]. In both cases, reversal potentials are hyperpolarized relative to the AP threshold value, suggesting a common cortical mechanism in which inhibitory neurons dynamically regulate the gain of fast sensory-evoked synaptic input. Specifically, inhibition clamps the synaptic response to values just below the AP threshold allowing precise control of sparse neuronal firing. Our results show that the removal of somatosensory input disrupts this fine balance, potentially through altered recruitment of cortical interneurons.

Although voltage clamp recordings could, in principle, provide a more direct way to address the synaptic mechanisms underlying the reach reversal potential, this approach is limited by substantial space errors. These errors are especially pronounced in neurons with extensive dendritic arbors, where incomplete control of the voltage can lead to a distorted estimate of synaptic conductances [80]. Alternative approaches will therefore be required to more accurately characterize the synaptic mechanisms driving the reach reversal potential.

### Correlations between M1 activity and ongoing forelimb movement is shaped by somatosensory input

Previous recording studies have shown that the spiking of M1 neurons is correlated with kinematic parameters of limb movement [81–84]. Our Vm recordings showed strong correlation with distance moved, weaker correlation with speed, and minimal correlation with acceleration (Fig 5), consistent with earlier extracellular recordings [85,86]. Importantly, removing somatosensory inputs changed both the timing and the magnitude of the correlation between Vm and forelimb distance. These results support a role for somatosensory input in the movement-related dynamics of M1 neurons, particularly the encoding of limb position during sustained movement.

### Outlook

The majority of studies of M1 function have used extracellular electrophysiological or optical recordings to monitor AP firing. However, spiking is determined by an integration of both synaptic input and intrinsic membrane properties. A mechanistic understanding of sensorimotor integration therefore requires direct measurement of Vm. To date, only a limited number of studies have recorded the Vm of motor cortical neurons during behavior using whole-cell recordings, including forelimb M1 [34,51], whisker M1 [87–89], and premotor cortex [36]. In the future, genetically encoded voltage sensors may enable more stable access to Vm activity [90,91].

Our study identified a central role of somatosensory input in the encoding of forelimb movement in the Vm of M1 neurons. While we used a nerve transection to remove somatosensory input, future work could build on the identification of new molecular markers for somatosensory afferent neuron subtypes [92] to dissect the relative contributions of proprioceptive compared to tactile inputs to M1 dynamics. Although somatosensory inputs have a significant role in M1 dynamics, a substantial amount of non-somatosensory synaptic input also contributes to M1 activity. Further work using cell-type and pathway-specific manipulations will be required to identify the contribution of inputs from other sensory and non-sensory pathways to M1 during behavior.

## Materials and methods

### Ethics statement

All experimental procedures were carried out in accordance with German animal welfare regulations and were approved by the Berlin animal welfare committee (Landesamt für Gesundheit und Soziales, license G0188/09).

### Surgery

P35-94 C57/bl6J mice were injected subcutaneously with metamizol (200 mg/ kg). At least 30 min later, anesthesia was induced with isoflurane (~1.5% in O_2_) until they were devoid of eye blink and paw withdrawal reflexes. Body temperature was monitored rectally and a heating pad was used for maintenance of body temperature at 37 °C. To evaluate the depth of anesthesia, eye blink and paw withdrawal reflexes as well as whisker movements were monitored. The eyes of the animal were protected against dehydration with ophthalmic ointment. The top of the head was shaved and the skull was exposed. The bone was gently cleaned with Ringer’s solution and 3% H_2_O_2_. A lightweight metal head support and a small plastic recording chamber, centered over the left-hand side forelimb motor cortex, was implanted onto the skull with glue and dental cement. Following surgery, mice were returned to their home cage for recovery and behavioral training. Mice received metamizol in the drinking water (~200 mg/kg). On the day of the experiment, a small craniotomy (~ 0.5 mm diameter) was drilled under isoflurane anesthesia at about +0.1 mm anterior, +1.5 mm lateral to Bregma (whole-cell recordings were made with shallow pipette angle to record underneath the intact skull). The dura was carefully removed and the exposed brain covered with the silicone elastomer Kwik Cast (WPI) to protect it from damage and dirt. Mice were then returned to their home cage to recover for at least 2 hours before recordings.

### Muscimol inactivation

Thin-walled glass micropipettes (tip diameter 10–20 µm) were backfilled with 1mM muscimol (Sigma) and 5% Pontamine Sky Blue (Sigma) in Ringer’s solution. During the experiment, trained (P46–P59) mice were head-fixed and the injection pipette slowly inserted to a depth of 700 µm from the cortical surface at the cortical target locations: either forelimb M1 (+ 0.5 mm anterior to Bregma, +1.7 mm lateral from midline), or auditory cortex (A1, −2.5 mm posterior to Bregma, +4 mm lateral from midline). A single 100 nl muscimol or control Ringer’s solution microinjection was made over approximately 1 min. The frequency of successful reaches, the duration of limb movement periods and grooming was recorded for 20 min from the end of injection and compared to a baseline session recorded on the previous day. Mice were then removed from the setup, returned to their home cage, and a recovery session was carried out the next day (S3B–S3E Fig). Post-hoc histology of the Pontamine Sky Blue location was used to confirm the location of the muscimol injection (S4 Fig).

### Intrinsic optical imaging

Mice were anesthetized with isoflurane (~1.5% in O_2_) and the representation of the right paw in the primary somatosensory forelimb cortex (fS1) was functionally located with intrinsic optical imaging. Briefly, the skull was illuminated with red light (630 nm) while the vibrotactile stimuli (10 Hz for 5 s) were applied to the right forepaw with a piezoelectric bimorph (PL 140.10, Physik Instrumente) using custom-written scripts in IGORpro (Wavemetrics, Portland, USA). The intrinsic signal was captured with a cooled monochrome Qicam CCD camera (Q-Imaging) and compared to the blood vessel pattern that had been imaged with green illumination (530 nm) to guide positioning at the surface of the cortex. Intrinsic imaging of the fS1 was used across all mice to help confirm the stereotactic position of the forelimb motor cortex.

### Forelimb intracortical microstimulation motor mapping

Male C57BL/6J mice (P46–P59) were implanted with a light-weight metal head-holder as described above. To locate forelimb M1, a craniotomy (3 mm anterior to 1.5 mm posterior, 0 to 3 mm lateral relative to Bregma) was made over forelimb somatosensory-motor cortex of the left hemisphere under isoflurane anesthesia (~2% in O_2_), with the dura left intact. To minimize cortical swelling, the brain was covered with 1.8% Agarose in Ringer’s solution containing, in mM: 135 NaCl, 5 KCl, 5 HEPES, 1.8 CaCl_2_, and 1 MgCl_2_. An Ag/AgCl ground electrode was placed in the recording chamber in contact with the Ringer’s solution. To map the forelimb motor area, a 0.5 MΩ tungsten microelectrode (Microprobe, MD, USA) was inserted vertically to a depth of 800 µm, targeting L5 neurons. Isoflurane anesthesia levels were reduced (~0.7% to 1% in O_2_) to evoke motor responses. Intracortical current pulse trains (30 ms duration containing 10 cathodal pulses, 0.4 ms pulse width, delivered at 333 Hz) were generated with an ITC18 analogue-to-digital converter controlled by IgorPro (Wavemetrics) and delivered via an A395R Linear Stimulus Isolator (World Precision Instruments). Stimulation sites were arranged in a grid pattern with 250 µm spacing. Stimulation currents ranged from 0 to 100 µA. To identify forelimb movements, current amplitude was increased in 10 µA steps until a movement was observed, then decreased to determine the threshold with a resolution of 0.5 µA. In two out of seven animals, both the CFA and the rostral forelimb area (RFA) [Neafsey and Sievert 1982] [93] were identified. Mapping along a given axis was stopped when stimulation at 250 and 500 µm from the last responsive site failed to evoke movement. Across animals, intrinsic imaging located the center of forelimb S1 at 0.05 mm rostral and 2.45 mm lateral to Bregma, while the CFA center was located at 0.3 mm rostral and 1.63 mm lateral to Bregma. This spatial relationship between forelimb S1 and M1 is consistent with our previous findings [21].

### Behavioral training

Mice were first habituated to the experimenter, handling, and the training setup. They were then habituated to head restraint with free access to water. Following habituation, mice were placed under water restriction and trained to perform a forelimb reaching task for a water reward. During training, mice used their right forepaw to move from a “rest” to “target” capacitance sensor (12 mm diameter, Pepperl and Fuchs), while their left paw rested on a custom-made support bar. The behavioral training setup was under the control of a custom-written software running on a Logo control system (Siemens). To discourage sustained contact with the target sensor, reward delivery was conditional on contacting the rest sensor before contact with the target sensor. In addition, once the target sensor registered a touch, it rapidly retracted away from the mouse on a horizontal pressure-controlled sliding rail system (SMC) and returned to its original position only after the paw was placed in contact with the rest sensor. A successful touch of the target sensor triggered the delivery of a ~5 µl water drop via a pressure gradient system controlled by a solenoid valve (Norgren). In some cases, mice pressed the target sensor a second time before it had retracted. These data were not included for further reach analysis. At the start of training, the target sensor was positioned close to the rest sensor (<1 cm). During supervised training, the target sensor was gradually moved further away, dependent on each animal’s performance, until mice reliably performed reaches of ~3 cm (center of rest sensor to center of target sensor). The duration and conditions of each training session depended on the motivation and performance of the individual mouse, with the aim of achieving a high rate of successful reaches on the day of electrophysiological recording.

### Sensory nerve transection procedure

In 14 implanted and trained mice, sensory nerves innervating the right forelimb were transected following the final training session. Under isoflurane anesthesia (~2% in O_2_), a hemilaminectomy was performed from spinal segments C4 to T2, followed by a transection of the dorsal roots from C5 to T1 (Figs 1C and S1A). To maintain hydration in water-restricted mice, we administered subcutaneous Ringer’s containing metamizol (200 mg/kg). During recovery, mice were kept in a heated cage with access to food pellets soaked in Ringer’s/metamizol. The behavioral/electrophysiological experiment were typically performed on the following day and only delayed if additional recovery time was required.

### Whole-cell patch-clamp recordings

Whole-cell patch-clamp recordings were performed in current-clamp mode using an Axon Multiclamp 700b amplifier (Molecular Devices). Recording electrodes were pulled from 2 mm borosilicate glass (Hilgenberg) and filled with intracellular solution containing (in mM): 135 K-gluconate, 4 KCl, 10 HEPES, 10 phosphocreatine, 4 MgATP, 0.3 Na3GTP (adjusted to pH 7.3 with KOH), and 2 mg/ml biocytin. An Ag/AgCl ground electrode was placed in the recording chamber filled with Ringer’s solution containing (in mM): 135 NaCl, 5 KCl, 5 HEPES, 1.8 CaCl_2_, 1 MgCl_2_. Recordings were filtered at 10 kHz and digitized at 20 kHz by an ITC 18 analogue to digital converter (Instrutech Corporation) under the control of IgorPro (Wavemetrics). The lowest 10% of Vm values in the Quiet state were used as an inclusion criterion. In 5 of 44 neurons, this value was between −55 mV and −49 mV, in all remaining neurons it was more negative than -55mV. The liquid junction potential was not subtracted from Vm values.

### Histology

Mice were deeply anesthetized by intraperitoneal injection of 10% urethane and transcardially perfused with 4% paraformaldehyde. Brains were removed, post-fixed in 4% paraformaldehyde and stored in phosphate buffer before histological processing. 100 µm thick coronal sections were cut using a Leica VT1000 S vibrating microtome. Slices were then stained for cytochrome oxidase and biocytin with a standard ABC kit (Vectastain) and DAB enhancement, then mounted in Moviol and stored at 4 °C. Labeled neurons were imaged, traced, and reconstructed using NeuroLucida (MicroBrightField). Cortical layers (L2/3 or L5) were determined based on histological inspection and relative cortical depth (S5 Fig).

### Tracking forelimb movement

One or more reflective markers (≤1 mm diameter, spherical markers from Loligo Systems ApS or custom markers made from 3M Scotchlite high reflection foil) were attached to the right forelimb using adhesive. Markers were illuminated with a ring of infrared LEDs surrounding a Planar 1,4/50 ZF-IR objective (Carl Zeiss). Forelimb movements were recorded at 500 Hz by an infrared high-speed imaging system (GS Vitec, Bad Soden Salmünster, Germany). Forelimb movements were tracked offline using the motion tracking plugin Blender (blender.org).

### Extraction of forelimb movement features

Forelimb kinematics were derived from the tracked X-Y position of the reflective marker. Forelimb position was defined as the absolute distance between the marker and the estimated center of the rest capacitive sensor. Forelimb speed was computed from the position coordinates using a Savitzky-Golay derivative filter (polynomial order 3, 9-sample sliding window), and combined using trigonometry to yield a one-dimensional (1D) absolute speed signal. Forelimb acceleration was derived by applying the same derivate filter to the 1D speed signal. From these 1D kinematic parameters (position, speed, and acceleration), we extracted the following behavioral features:

1. *Reaches* were defined as movements in which the forepaw remained within 1 cm of the rest sensor for at least 1 s, followed by a movement that crossed the 1 cm distance threshold (brown ticks, Fig 1D and 1E).
2. *Movement onsets* from quiet (Fig 1J) were detected by analyzing the absolute speed. Quiet periods were identified as 1 s windows during which speed exceeded 1 cm/s for less than 1% of the time, immediately followed by a 1 s window in which speed exceeded 1 cm/s for more than 50% of the time.
3. *Quiet and Move periods* across the entire session were identified using a sliding 1 s window to the speed measurement. Time points were labeled as “Quiet” when the forelimb speed was greater than 1 cm/s in less than 5% of the 1 s window, and as “Move” when it exceeded this threshold for more than 50% of the window.

### Analysis of neuronal activity

All data analysis was performed in MATLAB (R2019b) using custom-written scripts. We developed a custom analysis library for all pre-processing steps. In the IT group of 21 mice, one neuron was recorded from 20 mice and two neurons were recorded from one mouse. In the NC group of 14 NC mice, one neuron was recorded from 8 NC mice, two neurons were recorded from 4 NC mice, and three neurons were recorded from 2 mice. Each recording was treated as an independent observation. Statistical analysis throughout the study is non-parametric tests including Wilcoxon signed-rank test for paired comparisons, Mann–Whitney *U* test for unpaired, as well the *Z* test to test the differences between proportions. Relationships between variables were assessed using linear regression, and computed Pearson correlation coefficients derived from these regressions. To assess potential bias from multiple recordings in individual mice, we repeated the main comparisons between IT and NC neurons (*p* values in the main figures) using a linear mixed-effects model (MATLAB function *fitlme*), grouping recordings by mouse. This analysis showed similar or stronger significance than the non-parametric tests reported in the manuscript, indicating that treating individual neurons as independent measurements did not inflate effect sizes or significance levels.

AP timing was extracted by thresholding a high-pass filtered signal (200 Hz cutoff). To remove the influence of APs on the analysis of the subthreshold Vm, the signal in a window from 10 ms before to 20 ms after an AP was excluded and replaced by NaN symbols.

To quantify the frequency content of the Vm (Fig 3J–3L), power spectra were computed using the MATLAB function *pspectrum* as well as power in the 1–5 Hz and 30–80 Hz bands.

The reach reversal potential was estimated as the Vm value at which the linear regression between baseline Vm (mean −1 to −0.25 s before reach onset) and reach response amplitude crossed zero.

The reach response amplitude is the difference between the mean Vm measured from 0.25 to 1 s after reach onset and the baseline Vm. The reach spiking response (Hz) was measured as a spiking frequency relative to baseline, using the same intervals as for the Vm reach response.

The AP threshold was estimated from the raw Vm around AP, and corresponded to the Vm value at the time of peak of the third derivative of the Vm in the 1 ms before the peak of the AP.

Neurons were classified as enhanced or suppressed based on their average Vm during Quiet and Move periods. Enhanced neurons showed a more depolarized Vm during Move compared to Quiet, whereas suppressed neurons showed the opposite pattern.

To classify the polarity of a neuron’s Vm modulation around touch times, we measured the sign of the Vm change in a 200 ms window centered around touch compared to a 200 ms window −300 to −100 ms prior to touch. We used a time window centered around touch because of the limited accuracy of the touch time estimate obtained from the capacitive sensor, even with additional control using the camera system.

## Supporting information

S1 Fig Cutting the forelimb somatosensory nerves abolishes somatosensory response in primary forelimb somatosensory cortex. (**A**) Intrinsic optical imaging response of the forelimb primary somatosensory cortex (fS1) to forelimb vibrotactile stimulation in mice with intact somatosensory nerves. Left: cortical surface vasculature imaged with green light. Right: peak intrinsic signal at the same cortical location in response to a vibrotactile stimulation of the forepaw. The cyan square marks the estimated peak of the intrinsic signal response. Each row corresponds to one of the seven mice tested. (**B**) Same as (A), but showing the intrinsic signal response in the same mice after forelimb somatosensory nerves cut. The cyan square is positioned at the same location as in (A), aligned using the blood vessel pattern.
(TIFF)

S2 Fig Additional analysis of forelimb movements in IT and NC mice. (**A**) Examples of forelimb movements in an IT (left) and a NC mouse (right). During Quiet periods, the forelimb position is stable in IT mice, whereas NC mice show a slow drift of forelimb position at speeds below the movement threshold. (**B**) Histograms of the forelimb position at rest (100 ms time window before reach onset) in IT (black) and NC (green) mice. **: Mann–Whitney *p* = 0.0011. (**C**) Histograms of the duration of Quiet epochs in IT and NC mice. *: Mann–Whitney *p* = 0.013. (**D**) Histograms of forelimb speed during Quiet epochs in IT and NC mice. ***: Mann–Whitney *p* = 3.2 × 10^−8^. (**E**) Same as **D**, but for Move epochs. n.s.: Mann–Whitney *p* = 0.058. (**F**) Histograms of reach distance in IT and NC mice. Mann–Whitney *p* = 0.1556. (**G**) Histograms of the amplitude of forelimb movement during reaches in IT and NC mice. Mann–Whitney *p* = 0.93. (**H**) Positive linear relationship between average speed and amplitude was preserved in IT (Pearson *r* = 0.61) and NC mice (Pearson *r* = 0.36). (**I**) Average time course of reaches within Quiet periods (dark shade) and Move periods (light shade). Left: IT neurons. Right: NC neurons. (**J**) Histograms of peak forelimb speed for reaches made during a movement (top) versus quiet (bottom) periods. No significant differences were observed for either IT (left, Mann–Whitney *p* = 0.74) or NC (right, Mann–Whitney *p* = 0.66) neurons. (**K**) Histograms of reach duration for reaches during Movement (top) compared to Quiet (bottom) periods. No significant differences were observed for IT (left, Mann–Whitney *p* = 0.31) and NC (right, Mann–Whitney *p* = 0.72) neurons. The data underlying this Figure panels BCDEFGJK can be found in S1 Data.
(TIFF)

S3 Fig Identification of the M1 forelimb area and impact on forelimb behavior. (**A**) Overlay of the cortical sites where intracortical microstimulation evoked forelimb movements in 7 mice, identifying forelimb M1. The open circle represents the location selected for whole-cell recordings based on the average position of the microstimulation sites that triggered forelimb movements in the caudal forelimb area (CFA). The open square marks the average location of forelimb somatosensory cortex (fS1) determined by intrinsic optical imaging. The proximity of CFA and fS1 are consistent with known cortical anatomy. The filled circle denotes Bregma. (**B**) Example forelimb movements across three behavioral sessions: baseline (top), following muscimol injection into M1 (middle), and during a recovery session on the subsequent day (bottom). (**C**) Impact of cortical inactivation on forelimb reaching. The frequency of reaches performed by 3 groups of mice either with muscimol in M1 (*n* = 5), Ringer’s in M1 (*n* = 3) or muscimol in auditory cortex (A1) (*n*= 3). Lines show data from individual mice. **: Mann–Whitney *p* = 0.0079. (**D**) Same as (C), but for the percentage of time spent performing any forelimb movement during the session. **: Mann–Whitney *p* = 0.0079. (**E**) Same as (C), but for face grooming.
(TIFF)

S4 Fig Anatomical confirmation of the muscimol injection site. (**A**) Example coronal slice from a mouse following injection of muscimol together with Pontamine Sky Blue into the CFA. Black outline shows injection site. (**B**) Reconstitution of the cortex area borders around the injection site, based on the Paxinos atlas.
(TIFF)

S5 Fig Anatomical and electrophysiological characterization of recorded neurons. (**A**) Examples of anatomically reconstructed L2/3 (gray) and L5 (black) neurons from intact (IT) mice. Histological identification of layer location was successful for 35 of 44 recordings. In the absence of histology, neurons recorded less than 400 µm from the pial surface based on micromanipulator depth were classified as L2/3, while deeper recordings were classified as L5. (**B**) Histogram showing depth of recorded neurons in IT mice and NC mice, estimated from anatomical reconstructions and depth estimates. Light colors show L2/3 neurons, dark colors L5 neurons. (**C**) Mean baseline Vm estimated from 10 s of data with hyperpolarized Vm for each neuron. Color coding is same as in (B). In IT mice, the baseline Vm was significantly more hyperpolarized in L2/3 than L5 neurons (Mann–Whitney *p* = 0.0039). In NC mice there was no significant difference (n.s.: Mann–Whitney *p* = 0.060). The data underlying this Figure panels BC can be found in S1 Data.
(TIFF)

S6 Fig Control analysis related to findings in Figs 5 and 2. (**A–E overview**) *Differences in Vm-forelimb distance correlation are not explained by differences in movement frequency content.* Forelimb distance is more strongly correlated with M1 Vm dynamics in IT mice than in NC mice (Fig 5E and 5H). One possibility is that this could be explained by differences in the frequency content of forelimb movements in IT compared to NC mice. To test this, we selected subsets of data with the same average power spectrum and assessed whether the correlations persist. (**A**) Average power-spectrum of forelimb distance across all movement periods in IT recordings (black) compared to NC recordings (green). Light background shows SEM across sessions. Limb position measurements were extracted during movement bouts longer than 1 s. Power spectra were calculated for individual bouts and averaged within sessions. (**B**) Same as (A), but for a subsets of movement bouts in IT and NC mice selected by matched power spectrum at 5 and 50 Hz. (**C**) Population average of the absolute cross-correlation of the Vm with forelimb distance for the matched subset of movement bouts. As in the full dataset (Fig 5E), absolute Vm-distance correlation was greater in IT compared to NC mice. (**D**) Realignment of the absolute cross-correlograms from (C) to their peaks, highlights the difference in correlation half-width between IT and NC mice (Fig 5H). (**E**) The half width of the cross correlations in (D) is significantly larger in NC than in IT mice (equivalent to Fig 5I). *: Mann–Whitney *p* = 0.0325. (**F–H overview**) *Differences in Vm movement onset timing are not driven by larger Vm responses in IT neurons.* The Vm *Z*-scores at movement onset were on average larger in IT than in NC neurons (Fig 2H), which could bias estimates of onset timing (Fig 2I). To control for this, we removed IT neurons with the largest Vm *Z*-scores until the mean *Z*-scores were similar (IT *n* = 15 neurons, NC *n*= 22), and recomputed the timing of the onset of the increase of the *Z*-score > 2.5. (**F**) Histogram of the Vm change around movement onset, measured as the difference between the average Vm measured in a 0.25 s window after movement onset versus a 0.5 s window starting 1 s before movement onset. Right: peak *Z*-score following movement onset. In the analysis presented in (G) and (H), all IT cells beyond the vertical dashed line are excluded. (**G**) Average *Z*-score around movement onset for the match subsets defined in (F). Peak *Z*-score of the two groups are similar, but earlier onset in IT neurons is visible. (**H**) Timing of the onset of Vm *Z* scores above the Z = 2.5 threshold for the selected IT subset (*n* = 15) compared to the full NC dataset (*n*= 22). The significant difference in onset timing reported in the main text remains significant after normalization (***: Mann–Whitney *p* = 7.3 × 10^−4^). The data underlying this Figure panels EFH can be found in S1 Data.
(TIFF)

S7 Fig Distribution of the firing rate of all neurons that fired more than 10 spikes aligned to movement onset and renormalized to their peak value. (**A**) Firing rates of neurons recorded in intact (IT) mice. (**B**) Firing rates of neurons recorded in nerve-cut (NC) mice.
(TIFF)

S1 Data Spreadsheet providing the individual datapoints for the histograms displayed in the main and supplementary figures. (XLSX)

## Abbreviations

AP: action potential
CFA: caudal forelimb area
RFA: rostral forelimb area
fS1: forelimb somatosensory cortex
M1: motor cortex
