# Correction: Somatosensory input drives membrane potential dynamics in motor cortex during voluntary limb movement

**DOI:** 10.1371/journal.pbio.3003828

**Published:** 2026-05-26

**Authors:** The PLOS Biology Staff

## Notice of Republication

This article was republished on May 14, 2026, to correct an error in the XML file, which didn’t appear the PDF. Dr. Luc Estebanez and Dr. James F. A. Poulet contributed equally to this work. This now appears in both versions.
