# Supplementary figures and images for: Somatosensory input drives membrane potential dynamics in motor cortex during voluntary limb movement
Source: PLoS Biol. 2026 Apr 17;24(4):e3003749. doi: 10.1371/journal.pbio.3003749 (PMC13089743; doi:10.1371/journal.pbio.3003749)

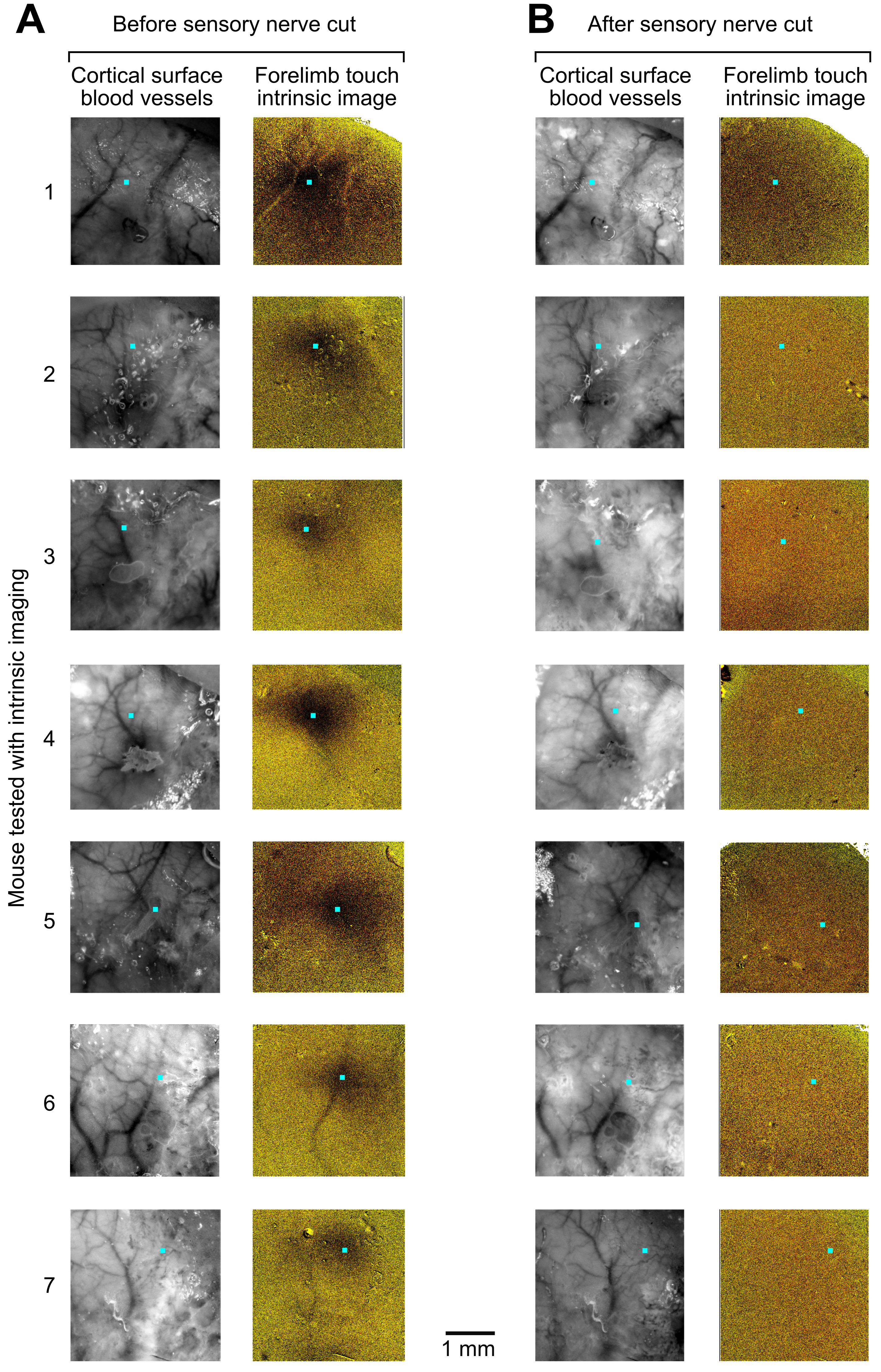

Supplement: S1 Fig — (A) Intrinsic optical imaging response of the forelimb primary somatosensory cortex (fS1) to forelimb vibrotactile stimulation in mice with intact somatosensory nerves. Left: cortical surface vasculature imaged with green light. Right: peak intrinsic signal at the same cortical location in response to a vibrotactile stimulation of the forepaw. The cyan square marks the estimated peak of the intrinsic signal response. Each row corresponds to one of the seven mice tested. (B) Same as (A), but showing the intrinsic signal response in the same mice after forelimb somatosensory nerves cut. The cyan square is positioned at the same location as in (A), aligned using the blood vessel pattern. (TIFF) [file pbio.3003749.s001.tiff]

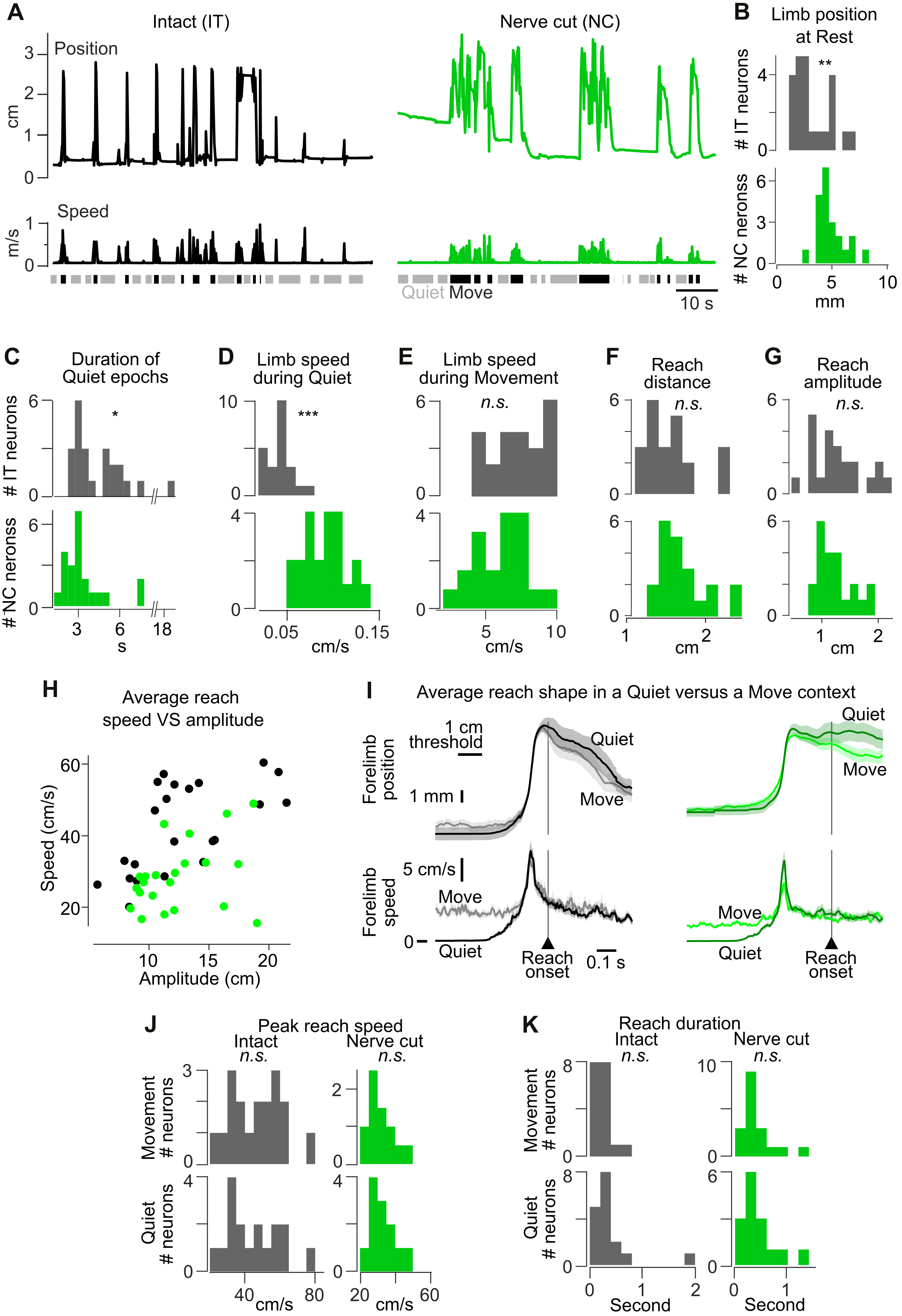

Supplement: S2 Fig — (A) Examples of forelimb movements in an IT (left) and a NC mouse (right). During Quiet periods, the forelimb position is stable in IT mice, whereas NC mice show a slow drift of forelimb position at speeds below the movement threshold. (B) Histograms of the forelimb position at rest (100 ms time window before reach onset) in IT (black) and NC (green) mice. **: Mann–Whitney p = 0.0011. (C) Histograms of the duration of Quiet epochs in IT and NC mice. *: Mann–Whitney p = 0.013. (D) Histograms of forelimb speed during Quiet epochs in IT and NC mice. ***: Mann–Whitney p = 3.2 × 10^−8^. (E) Same as D, but for Move epochs. n.s.: Mann–Whitney p = 0.058. (F) Histograms of reach distance in IT and NC mice. Mann–Whitney p = 0.1556. (G) Histograms of the amplitude of forelimb movement during reaches in IT and NC mice. Mann–Whitney p = 0.93. (H) Positive linear relationship between average speed and amplitude was preserved in IT (Pearson r = 0.61) and NC mice (Pearson r = 0.36). (I) Average time course of reaches within Quiet periods (dark shade) and Move periods (light shade). Left: IT neurons. Right: NC neurons. (J) Histograms of peak forelimb speed for reaches made during a movement (top) versus quiet (bottom) periods. No significant differences were observed for either IT (left, Mann–Whitney p = 0.74) or NC (right, Mann–Whitney p = 0.66) neurons. (K) Histograms of reach duration for reaches during Movement (top) compared to Quiet (bottom) periods. No significant differences were observed for IT (left, Mann–Whitney p = 0.31) and NC (right, Mann–Whitney p = 0.72) neurons. The data underlying this Figure panels BCDEFGJK can be found in S1 Data. (TIFF) [file pbio.3003749.s002.tiff]

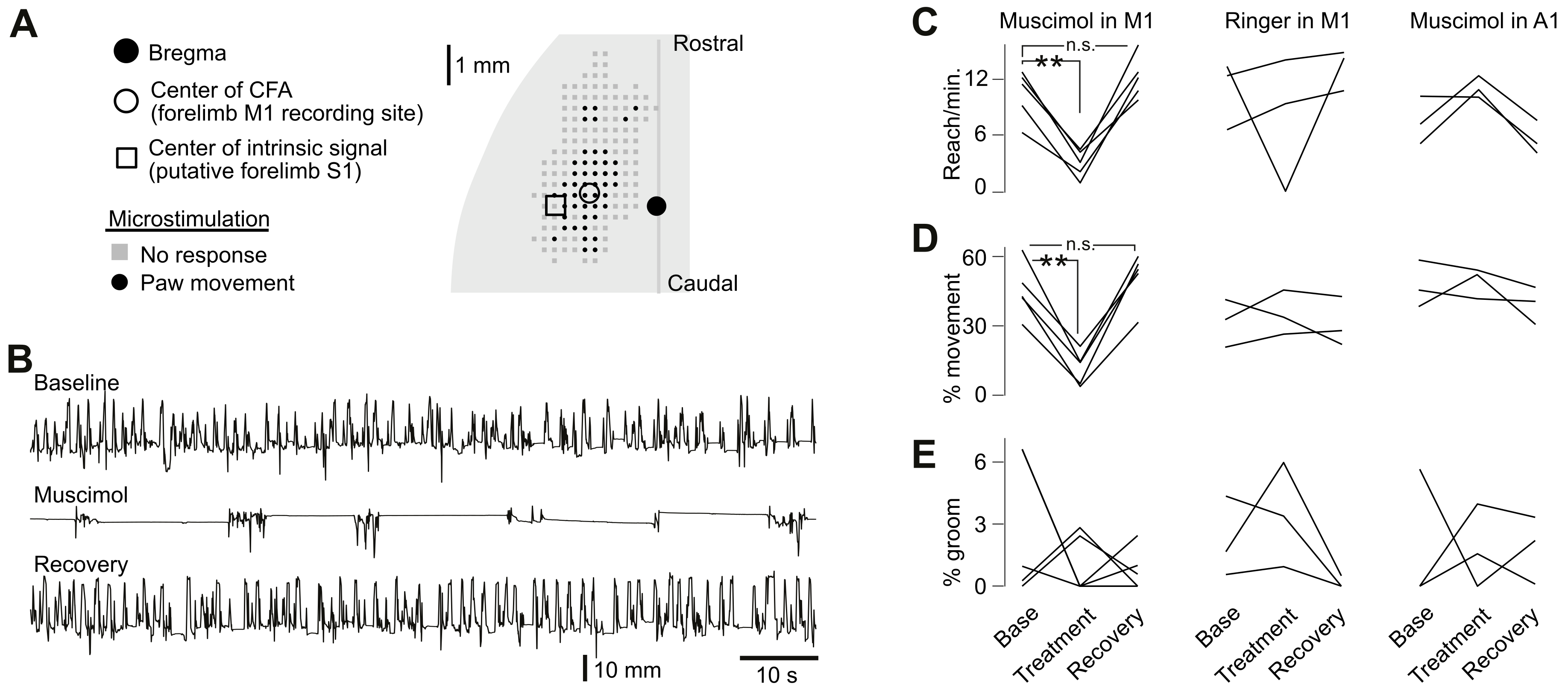

Supplement: S3 Fig — (A) Overlay of the cortical sites where intracortical microstimulation evoked forelimb movements in 7 mice, identifying forelimb M1. The open circle represents the location selected for whole-cell recordings based on the average position of the microstimulation sites that triggered forelimb movements in the caudal forelimb area (CFA). The open square marks the average location of forelimb somatosensory cortex (fS1) determined by intrinsic optical imaging. The proximity of CFA and fS1 are consistent with known cortical anatomy. The filled circle denotes Bregma. (B) Example forelimb movements across three behavioral sessions: baseline (top), following muscimol injection into M1 (middle), and during a recovery session on the subsequent day (bottom). (C) Impact of cortical inactivation on forelimb reaching. The frequency of reaches performed by 3 groups of mice either with muscimol in M1 (n = 5), Ringer’s in M1 (n = 3) or muscimol in auditory cortex (A1) (n= 3). Lines show data from individual mice. **: Mann–Whitney p = 0.0079. (D) Same as (C), but for the percentage of time spent performing any forelimb movement during the session. **: Mann–Whitney p = 0.0079. (E) Same as (C), but for face grooming. (TIFF) [file pbio.3003749.s003.tiff]

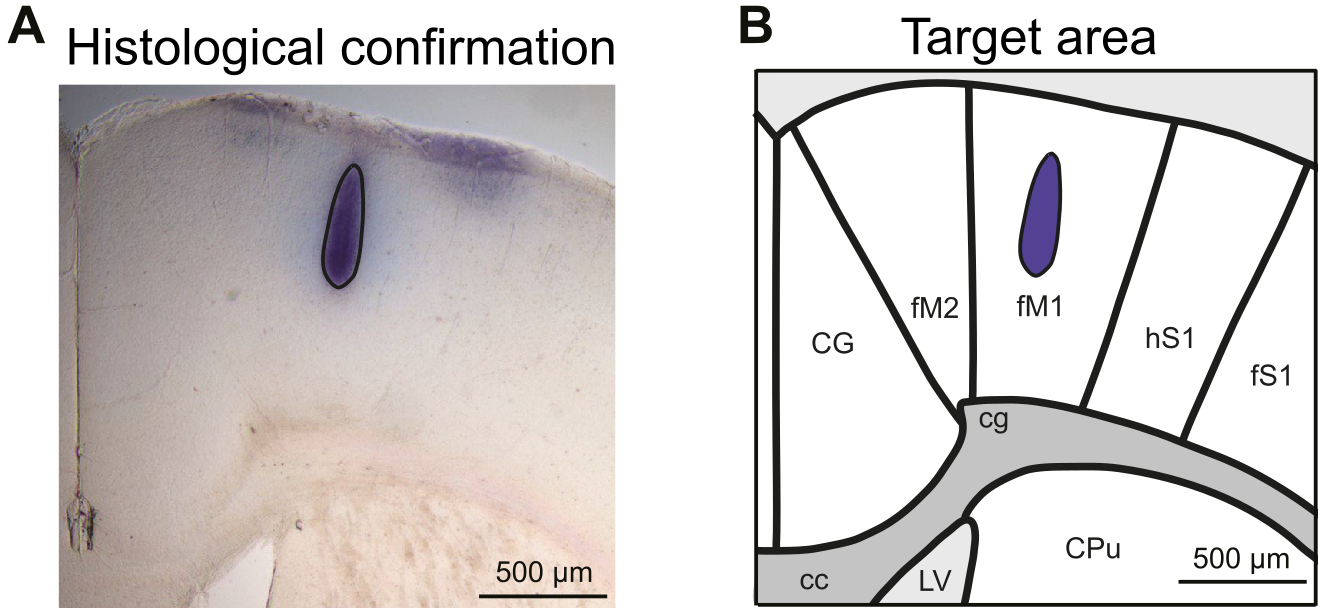

Supplement: S4 Fig — (A) Example coronal slice from a mouse following injection of muscimol together with Pontamine Sky Blue into the CFA. Black outline shows injection site. (B) Reconstitution of the cortex area borders around the injection site, based on the Paxinos atlas. (TIFF) [file pbio.3003749.s004.tiff]

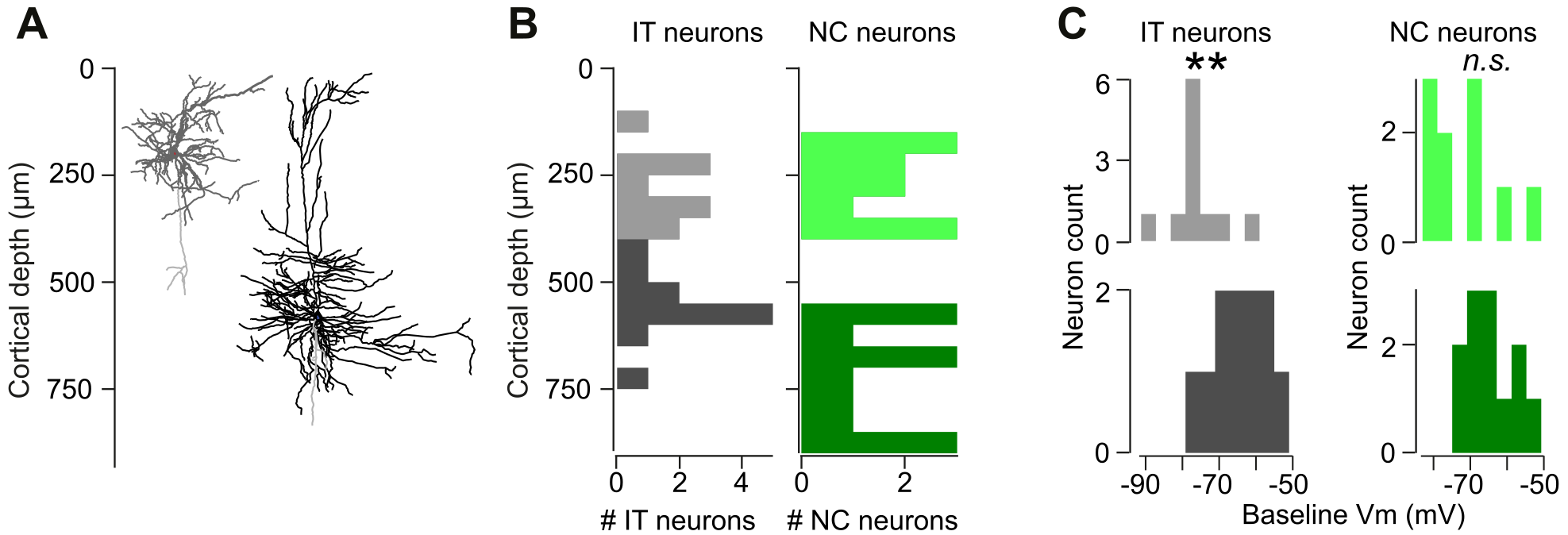

Supplement: S5 Fig — (A) Examples of anatomically reconstructed L2/3 (gray) and L5 (black) neurons from intact (IT) mice. Histological identification of layer location was successful for 35 of 44 recordings. In the absence of histology, neurons recorded less than 400 µm from the pial surface based on micromanipulator depth were classified as L2/3, while deeper recordings were classified as L5. (B) Histogram showing depth of recorded neurons in IT mice and NC mice, estimated from anatomical reconstructions and depth estimates. Light colors show L2/3 neurons, dark colors L5 neurons. (C) Mean baseline Vm estimated from 10 s of data with hyperpolarized Vm for each neuron. Color coding is same as in (B). In IT mice, the baseline Vm was significantly more hyperpolarized in L2/3 than L5 neurons (Mann–Whitney p = 0.0039). In NC mice there was no significant difference (n.s.: Mann–Whitney p = 0.060). The data underlying this Figure panels BC can be found in S1 Data. (TIFF) [file pbio.3003749.s005.tiff]

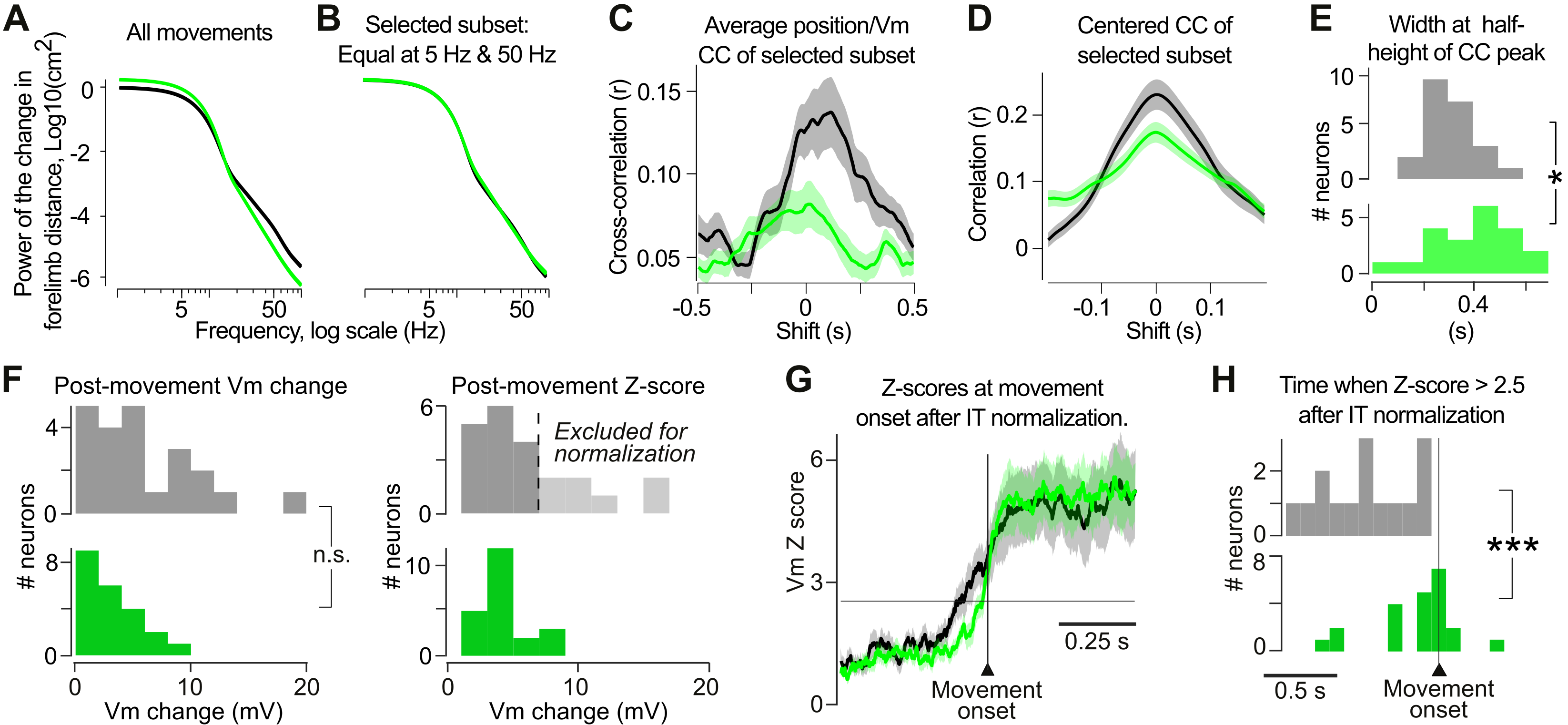

Supplement: S6 Fig — (A–E overview) Differences in Vm-forelimb distance correlation are not explained by differences in movement frequency content. Forelimb distance is more strongly correlated with M1 Vm dynamics in IT mice than in NC mice (Fig 5E and 5H). One possibility is that this could be explained by differences in the frequency content of forelimb movements in IT compared to NC mice. To test this, we selected subsets of data with the same average power spectrum and assessed whether the correlations persist. (A) Average power-spectrum of forelimb distance across all movement periods in IT recordings (black) compared to NC recordings (green). Light background shows SEM across sessions. Limb position measurements were extracted during movement bouts longer than 1 s. Power spectra were calculated for individual bouts and averaged within sessions. (B) Same as (A), but for a subsets of movement bouts in IT and NC mice selected by matched power spectrum at 5 and 50 Hz. (C) Population average of the absolute cross-correlation of the Vm with forelimb distance for the matched subset of movement bouts. As in the full dataset (Fig 5E), absolute Vm-distance correlation was greater in IT compared to NC mice. (D) Realignment of the absolute cross-correlograms from (C) to their peaks, highlights the difference in correlation half-width between IT and NC mice (Fig 5H). (E) The half width of the cross correlations in (D) is significantly larger in NC than in IT mice (equivalent to Fig 5I). *: Mann–Whitney p = 0.0325. (F–H overview) Differences in Vm movement onset timing are not driven by larger Vm responses in IT neurons. The Vm Z-scores at movement onset were on average larger in IT than in NC neurons (Fig 2H), which could bias estimates of onset timing (Fig 2I). To control for this, we removed IT neurons with the largest Vm Z-scores until the mean Z-scores were similar (IT n = 15 neurons, NC n= 22), and recomputed the timing of the onset of the increase of the Z-score > 2.5. (F) Histogram of t [file pbio.3003749.s006.tiff]

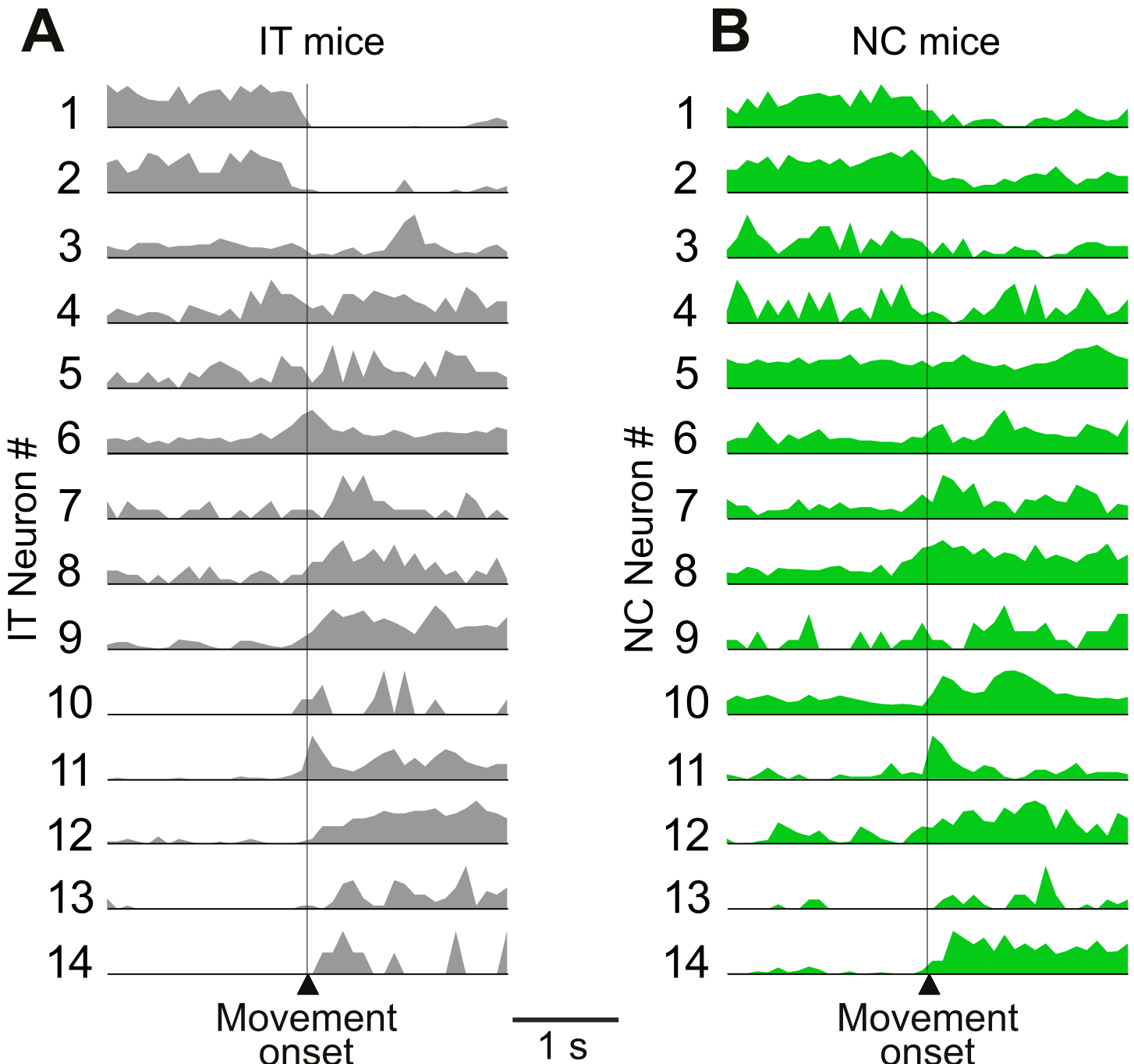

Supplement: S7 Fig — (A) Firing rates of neurons recorded in intact (IT) mice. (B) Firing rates of neurons recorded in nerve-cut (NC) mice. (TIFF) [file pbio.3003749.s007.tiff]
